# Phosphorylation of IDH1 Facilitates Progestin Resistance in Endometrial Cancer

**DOI:** 10.1002/advs.202310208

**Published:** 2024-04-06

**Authors:** Jingjie Li, Zuoshu Qin, Yunqi Li, Baozhu Huang, Qimeng Xiao, Peiqin Chen, Yifan Luo, Wenxin Zheng, Tao Zhang, Zhenbo Zhang

**Affiliations:** ^1^ Precision Research Center for Refractory Diseases Shanghai General Hospital Shanghai Jiao Tong University School of Medicine Shanghai 201620 China; ^2^ Shanghai Institute of Hematology State Key Laboratory of Medical Genomics National Research Center for Translational Medicine Ruijin Hospital Shanghai Jiao Tong University School of Medicine Shanghai 200025 China; ^3^ Department of Pathology University of Texas Southwestern Medical Center Dallas TX 75390 USA; ^4^ Department of Obstetrics and Gynecology University of Texas Southwestern Medical Center Dallas TX 75390 USA; ^5^ Simon Comprehensive Cancer Center University of Texas Southwestern Medical Center Dallas TX 75390 USA; ^6^ Department of Orthopedics Shanghai General Hospital Shanghai Jiao Tong University School of Medicine Shanghai 200080 China; ^7^ Reproductive Medicine Center Department of Obstetrics and Gynecology Tongji hospital School of Medicine Tongji University Shanghai 200065 China

**Keywords:** endometrial cancer, IDH1 phosphorylation, p38, progestin resistance

## Abstract

The progestin regimen is one of the main therapeutic strategies for women with endometrial cancer who undergo conservative management. Although many patients respond well to initial therapy, progestin‐refractory disease inevitably emerges, and the molecular basis underlying progestin resistance has not been comprehensively elucidated. Herein, they demonstrated progestin results in p38‐dependent IDH1 Thr 77 phosphorylation (pT77‐IDH1). pT77‐IDH1 translocates into the nucleus and is recruited to chromatin through its interaction with OCT6. IDH1‐produced α‐ketoglutarate (αKG) then facilitates the activity of OCT6 to promote focal adhesion related target gene transcription to confer progestin resistance. Pharmacological inhibition of p38 or focal adhesion signaling sensitizes endometrial cancer cells to progestin in vivo. The study reveals p38‐dependent pT77‐IDH1 as a key mediator of progestin resistance and a promising target for improving the efficacy of progestin therapy.

## Graphical abstract

The study reports a previously unrecognized link between IDH1 T77 phosphorylation and progestin resistance in endometrial cancer. Progestin treatment leads to p38‐dependent phosphorylation of IDH1 at threonine 77 (pT77‐IDH1). pT77‐IDH1‐induced focal adhesion signaling creates therapeutic vulnerabilities in progestin resistance, and pharmacological blockage of pT77‐IDH1 or focal adhesion has the potential to be translatable into the clinical setting.

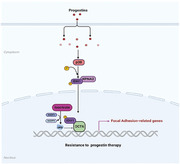

## 1 Introduction

Endometrial cancer (EC) is one of the most common gynecological cancers, and its prevalence has been significantly increasing.^[^ ^1^ ^]^ The standard treatment for EC involves hysterectomy and bilateral salpingo‐oophorectomy (THBSO) with or without lymphadenectomy, followed by further adjuvant therapy.^[^ ^2^ ^]^ Although EC is conventionally considered as a disease in postmenopausal women, ≈20% of women with EC are premenopausal, and 5% are less than 40 years of age.^[^ ^3^ ^]^ A growing number of reproductive‐age women are delaying child‐bearing and an increase in the frequency of EC among nulliparous women has been observed.^[^ ^4^ ^]^ As EC is frequently symptomatic at an early stage, most young women are diagnosed at stage I, which is still confined to the uterus.^[^ ^5^ ^]^ In such cases, standard surgery may compromise fertility, and a conservative approach has become a crucial component of fertility preservation care.

Fertility preservation management involves high‐dose oral progestins and levonorgestrel‐releasing intrauterine devices (LNG‐IUDs).^[^ ^3^, ^6^ ^]^ At present, the most extensively used progestins for conservative management are medroxyprogesterone acetate (MPA) and megestrol acetate (MA).^[^ ^7^ ^]^ Previous studies highlight a 50% to 70% overall response rate for patients receiving high‐dose oral progestin regimens, while others fail to respond to hormonal therapy. In parallel, some patients who initially responded relapse while on hormonal therapy.^[^ ^8^ ^]^ Despite the clinical use of progestins for decades, the mechanisms underlying its therapeutic resistance remain poorly understood.^[^ ^3^, ^9^ ^]^ Researchers have attempted to assess epithelial progesterone receptor (PR) isoforms (PRA and PRB) as determinants of progestin therapy resistance with limited success.^[^ ^10^ ^]^ Several studies have reported the involvement of the stromal PR axis in progestin resistance.^[^ ^9^ ^]^ There is also evidence that aberrant PI3K/AKT and Nrf2 signaling are associated with progestin therapy sensitivity.^[^ ^11^ ^]^ However, conclusively determining the key nodes that control progestin resistance is a major challenge. This highlights the critical need to identify of novel predictive factors for improved progestin response.

Cancer cells reprogram their energy metabolism to support bioenergetics and macromolecular synthesis, which ultimately promote tumor accumulation and dissemination and favor their defense against stress.^[^ ^12^ ^]^ Mounting evidence indicates that signal transduction pathways activated by oncogenic alterations in conjunction with factors in the tumor microenvironment facilitate metabolic reprogramming in cancer cells.^[^ ^13^ ^]^ A prevailing view posits that a key function of signal transduction components is to increase metabolic enzyme and metabolism‐related transcription factor activities.^[^ ^14^ ^]^ It has become increasingly clear that aberrantly regulated metabolic enzymes and their oncometabolites may serve as signaling modulators and contribute to tumor expansion and therapy resistance.^[^ ^15^ ^]^

Isocitrate dehydrogenase 1 (IDH1) is one of three isozymes (IDH1, IDH2, IDH3) which catalyze the oxidative decarboxylation of isocitrate (ICT) and produce α‐ketoglutarate (αKG) in the tricarboxylic acid (TCA) cycle.^[^ ^16^ ^]^ It functions as homodimers which reduce nicotinamide adenine dinucleotide phosphate (NADP+).^[^ ^16^ ^]^ Unlike the other two mitochondrial isozymes, IDH1 is primarily located in the cytosol.^[^ ^17^ ^]^ Cytoplasmic IDH1 plays an essential role in providing the reducing power (NADPH) for lipid synthesis and redox balance.^[^ ^18^ ^]^ As a key intermediate in the TCA cycle and glutamine decomposition, IDH1‐produced αKG acts as a nitrogen transporter and enables the activity of αKG‐dependent dioxygenases including methyl cytosine dioxygenase TET1 and histone demethylase JmjC, thus regulating the DNA hydroxymethylation and histone methylation status of the gene promoter.^[^ ^19^ ^]^ Oncogenic mutations in IDH1 have been identified in some malignancies, such as acute myeloid leukemias (AML) and lower‐grade and secondary glioblastoma (GBM).^[^ ^20^ ^]^ Many studies have focused on IDH mutations, and thus prompting the discovery of mutated IDH1 inhibitors. An example is the IDH1‐mutant inhibitor ivosidenib (AG‐120), which has been approved by the US Food and Drug Administration for the treatment of refractory acute myeloid leukemia.^[^ ^21^ ^]^ Recently, wild‐type IDH1 has been shown to play a role in tumor development and progression. IDH1 inhibition has been proved to enhance the effects of classic cancer therapies in several types of cancer by decreasing αKG, ATP, lipid, and dNTP synthesis and increasing ROS, lipid peroxidation, and protein oxidation, all of which are closely related to the IDH1 enzymatic activities.^[^ ^22^ ^]^ These findings suggest that activated IDH1 may mitigate the damage caused by stress that increases cell death and that inhibition of IDH1 activity may have therapeutic potential in some diseases, including cancer. Therefore, evaluation of IDH1 activity in cancer management is likely to lead to improved modalities. Notably, wild‐type IDH1 is enriched in endometrial cancer, whereas IDH1 mutations are rare.^[^ ^23^ ^]^ Our previous work reported that the aberrant expression of IDH1 lowers the therapeutic response to chemotherapy in endometrial cancer^[^ ^23^ ^]^; however, the functional consequences of IDH1 activity in endometrial cancer and its effect on the therapeutic response to progestin therapy are yet to be explored.

In this study, we identified a mechanism of progestin resistance mediated by p38‐dependent phosphorylation of IDH1 at threonine 77. We provide evidence that IDH1 T77 phosphorylation (pT77‐IDH1) is induced by p38 in response to progestin in endometrial cancer cells, which enhances IDH1 enzymatic activity and facilitates its nuclear redistribution. In the nucleus, IDH1 is recruited to chromatin through its interaction with transcription factor OCT6. IDH1‐produced α‐ketoglutarate (αKG) then facilitates the activity of OCT6 to promote focal adhesion related targets transcription, which further confers progestin resistance. Pharmacological inhibition of p38 or focal adhesion signaling restores the vulnerability of refractory endometrial cancer to progestin therapy in preclinical mouse models. Our findings suggest that p38‐dependent pT77‐IDH1 is a key mediator of progestin resistance and provide a therapeutic strategy to restore the progestin response in endometrial cancer.

## 2 Results

### 2.1 p38‐Mediated Phosphorylation of IDH1 at Threonine 77 is Induced in Response to Progestin Treatment

**Figure 1:**
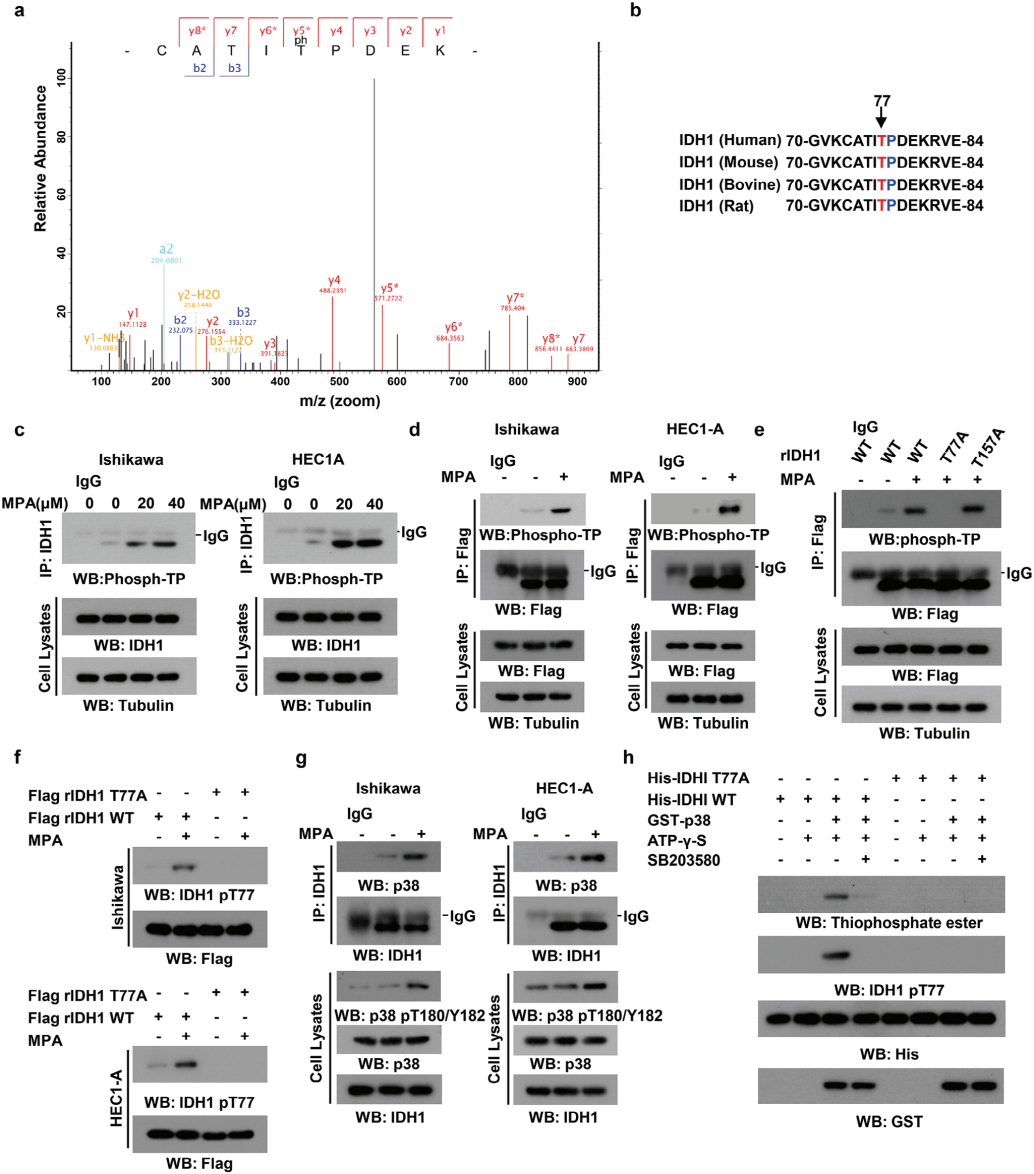
MPA induces p38‐dependent IDH1 T77 phosphorylation. a) Ishikawa cells overexpressing Flag‐tagged IDH1 were treated with 40 µM MPA or control DMSO. 24 h post treatment, cells were harvested and immunoprecipitation assays were performed using Flag‐M2 beads. The immunoprecipitates were analyzed by mass spectrometry. Tandem mass spectrometry spectrum analysis of a tryptic IDH1 73‐CATITPDEK‐81fragment at m/z 558.23 indicated the phosphorylated T77 in MPA‐treated group. The scan number was 11219, and the score was 71.03. b) Sequence alignment of the IDH1 peptide (amino acid residues 70 to 84) across various species. T77 of IDH1 is evolutionarily conserved in the indicated species and highlighted in red. The proline located in the T‐P motif is highlighted in blue. c) Ishikawa and HEC‐1A cells were cultured in the presence or absence of indicated concentrations of MPA for 24 h. Co‐immunoprecipitation analyses were performed using the anti‐IDH1 antibody. d) Flag‐IDH1 was immunoprecipitated followed by immunoblotting with an anti‐Phosphothreonine‐Proline antibody. e) Ishikawa cells stably expressing shRNA‐resistant (r) WT IDH1, IDH1 T77A or IDH1 T157A mutants were treated with or without 40 µm MPA for 24 h, and co‐immunoprecipitation analyses were performed with indicated antibodies. f) Ishikawa and HEC1‐A cells stably expressing shRNA‐resistant (r) WT IDH1or IDH1 T77A mutant were treated with 40 µm MPA or left untreated for 24 h. The phosphorylated T77‐IDH1 was detected by immunoblotting with an anti‐IDH1 pT77 antibody. g) Ishikawa and HEC‐1A cells were treated with or without 40 µM MPA for 24 h. Co‐immunoprecipitation analyses with indicated antibody were performed. h) Purified p38 was mixed with the indicated bacterially purified His‐IDH1 proteins and ATP‐γ‐S in the presence or absence of the p38 inhibitor SB203580. Immunoblotting was performed with the indicated antibodies.

The activity regulation of metabolic enzymes often results from their own posttranslational modifications, including phosphorylation,^[^ ^24^ ^]^ acetylation,^[^ ^25^ ^]^ ubiquitination,^[^ ^26^ ^]^ and malonylation.^[^ ^27^ ^]^ To identify the posttranslational modifications of IDH1 in response to progestin therapy, we performed co‐immunoprecipitation experiments, followed by mass spectrometric profiling of Ishikawa cells overexpressing Flag‐tagged IDH1 in the presence or absence of MPA. After fragmentation using trypsin, mass spectrometry analysis identified a phosphorylated fragment matched to the peptide 73‐CATITPDEK‐81 in MPA‐treated group, indicating threonine 77 (T77) was phosphorylated (**Figure** **1**a); and this residue is highly conserved among vertebrate IDH1 orthologs (Figure 1b). Given phosphorylation on threonine 77 preceding a proline (pT‐P), we further explored IDH1 threonine phosphorylation induced by MPA using an anti‐phosphothreonine‐proline antibody. MPA treatment increased the phosphorylation of endogenous (Figure 1c) and exogenous (Figure 1d) IDH1 in Ishikawa and HEC1‐A endometrial cancer cells. Furthermore, analysis of the IDH1 protein sequence revealed two threonine residues (T77 and T157), with their localization as part of the T–P motifs (Figure 1b; Figure S1a, Supporting Information). We then depleted IDH1 in endometrial cancer cells and rescued these cells with Flag‐tagged shRNA‐resistant (r) IDH1 wild‐type (WT), T77A and T157A (Figure S1b, Supporting Information), and found that only the T77A mutation failed to be phosphorylated after MPA administration (Figure 1e). Consistently, an antibody generated against pT77‐IDH1 (Figure S1c, Supporting Information) recognized phosphorylated wild‐type (WT) IDH1 but not the T77A mutant in Ishikawa and HEC1‐A cells (Figure 1f; Figure S1d, Supporting Information). Similarly, megestrol acetate (MA), another commonly used progestin for conservative management, promoted IDH1 T77 phosphorylation (Figure S1e, Supporting Information), implying that progestin exposure induces pT77‐IDH1 in EC cells. We next sought to search for the upstream signals responsible for IDH1 T77 phosphorylation in response to MPA treatment. We used the online tool NetPhos‐3.1 (https://services.healthtech.dtu.dk/service.php?NetPhos‐3.1) and found that p38MAPK (p38) had the highest NetPhos score (Figure S1f, Supporting Information) as a potential kinase that may promote IDH1 phosphorylation at T77. The stress‐responsive kinase p38 was activated by MPA in Ishikawa and HEC1‐A cells (Figure S1g, Supporting Information). In addition, p38 physically interacted with IDH1 in Ishikawa and HEC1‐A cells exposed to MPA (Figure 1g). Genetic or pharmacological inhibition of p38 impaired the MPA‐induced IDH1 T77 phosphorylation (Figure S1h,i, Supporting Information). An in vitro kinase assay showed that p38 could phosphorylate recombinant WT IDH1, instead of the T77A mutant, and this reaction was blocked by the p38 inhibitor, SB203580 (Figure 1h). Together, these findings suggest that MPA induces p38‐dependent phosphorylation of IDH1 at threonine 77.

### 2.2 Phosphorylation at T77 Enhances IDH1 Enzymatic Activity

**Figure 2:**
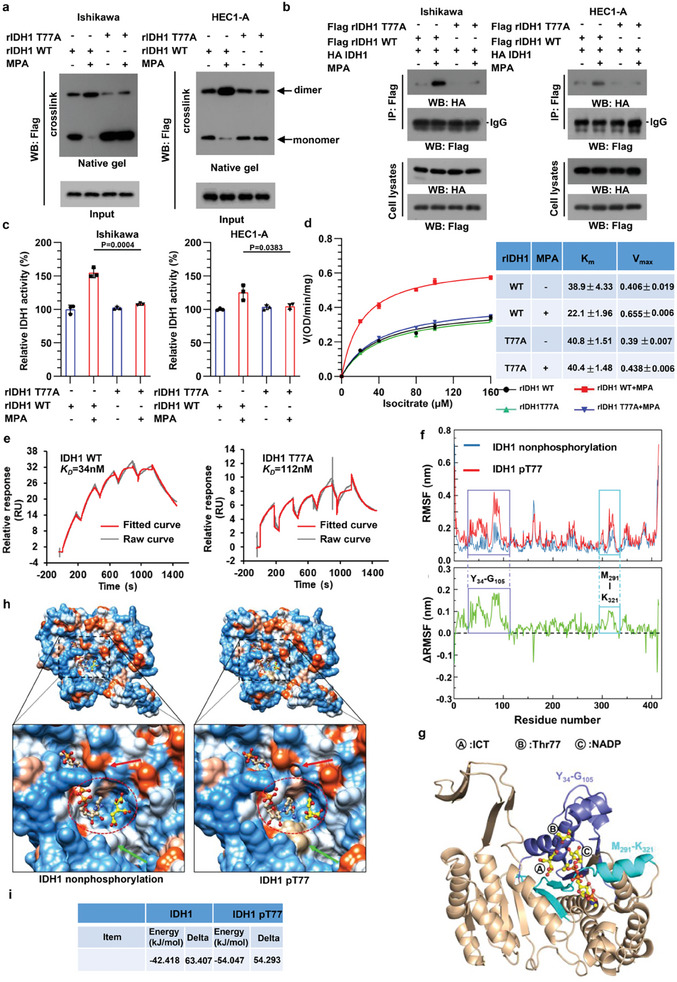
MPA‐mediated IDH1 T77 phosphorylation results in increased homodimerization, substrate binding and enhanced enzyme activity of IDH1. a) Ishikawa and HEC1‐A cells harboring WT IDH1 or its T77A mutant were cultured upon MPA exposure. Extracts were then treated with or without 0.025% glutaraldehyde and analyzed by immunoblotting. Dimer (upper) and monomer (lower) were indicated by black arrows. b) Ishikawa and HEC1‐A cells expressing Flag‐tagged WT IDH1 or IDH1 T77 mutant were co‐expressed with HA‐tagged WT IDH1. The interaction between differently tagged proteins was determined by immunoprecipitation. c) Ishikawa and HEC1‐A cells expressing WT IDH1 or its T77A mutant were treated with or without MPA. Flag‐tagged proteins were purified by Flag‐M2 beads. IDH1 activity was measured using the purified proteins by an IDH1 activity kit. The values are presented as mean ±SD (n = 3). An unpaired 2‐tailed Student's t test was used. d) Flag‐ tagged IDH1 proteins precipitated in (c) were used to detect the Vmax and Km of IDH1. e) Surface plasmon resonance analyses of recombinant IDH1 WT and T77A protein affinities to ICT. Red curves show the fitting to the raw data (shown in grey) using a 1:1 binding model. f) The RMSF and ΔRMSF of IDH1 with or without T77 phosphorylation were shown. g) Structure of T77 phosphorylated IDH1 in complex with ICT and NADP. h) Structural view of non‐phosphorylated and T77‐phosphorylated IDH1 generated from molecular dynamic simulation. Red ovals indicate the active site cleft. The bound NADP (in tan) and isocitrate (in gold) are shown as ball‐and‐stick models. Red and green arrows indicate the structural variation in two states. i) The binding energy of non‐phosphorylated and T77‐phosphorylated IDH1 to ICT.

In an attempt to investigate the role of T77 in the regulation of IDH1, we compared the activity of WT IDH1 with that of the T77A mutant in several assays. IDH1 exists as a mixture of monomers and dimers but functions as a homodimer.^[^ ^16^ ^]^ A glutaraldehyde cross‐linking assay showed that MPA treatment led to increased dimer formation from IDH1 monomers in Ishikawa and HEC1‐A cells expressing the wild‐type, but not the T77A mutant IDH1 (**Figure** **2**a). We next determined the interaction between Flag‐ and HA‐tagged IDH1 in EC cells. The T77A mutation largely blocked the association of the differently tagged IDH1 induced by MPA (Figure 2b). This was further confirmed by incubation of MPA with EC cells harboring WT or T77A mutated IDH1, where MPA was only able to increase NADPH and αKG levels in EC cells with WT IDH1(Figure S2a,b, Supporting Information). Furthermore, we treated Ishikawa cells with or without MPA and purified the wild‐type (WT) IDH1 and T77A proteins by immunoprecipitation. We then reconstituted an in vitro IDH1 enzymatic reaction and found that compared to its WT counterpart, the IDH1 T77A mutant significantly impaired MPA‐induced IDH1 enzymatic activity (Figure 2c). Further kinetic studies demonstrated that as the concentrations of isocitrate (ICT) increased, phosphorylation of WT IDH1, but not the T77A mutant by MPA yielded a much higher maximum reaction rate (Vmax) and decreased Michaelis‐Menten constant (Km) values for ICT (Figure 2d). Consistently, the binding affinities between IDH1 variant proteins and ICT were assessed by surface plasmon resonance (SPR) assays and the results indicated that WT IDH1 exhibited a lower dissociation constant (KD) value for ICT than did the phosphorylation inactivation IDH1 T77A mutant; and the phosphomimic IDH1 T77D mutant had the smallest KD value, suggesting that ICT prefers to bind to IDH1 in the phosphorylated state (Figure 2e; Figure S2c,d, Supporting Information). IDH1 exists in an asymmetric open conformation when bound to NADP. Upon rapid diffusion of ICT into the binding sites, the conformation transits to a symmetric closed one (PDB: 1T0L), wherein ICT interacts with the side chains of T77, S94, R100, R109, R132, Y139, and D275.^[^ ^28^ ^]^ We thus hypothesized that T77 phosphorylation may enhance IDH1 enzymatic activity by increasing substrate binding. We performed molecular dynamics (MD) simulations in a closed conformation and explored the binding of non‐phosphorylated and T77‐phosphorylated IDH1 to ICT. The results showed that although non‐phosphorylated IDH1 remained stable, phosphorylated T77‐IDH1 adopted a more dynamic conformation with an increase in the root mean square deviation (RMSD) and radius of gyration (Rg) starting from 15ns (Figure S2e, Supporting Information). We also found that IDH1 T77 phosphorylation promoted solvent accessible surface area (SASA) expansion which may favor binding to its substrate (Figure S2f, Supporting Information). Amino acid flexibility in non‐phosphorylated and T77‐phosphorylated IDH1 was shown as the root mean square fluctuation (RMSF) in Figure 2f. Amino acid fluctuations (ΔRMSF>0.1 nm) appeared in two loops (Y34‐G105 and M291‐K321) in T77‐phosphorylated IDH1 instead of non‐phosphorylated IDH1(Figure 2f). As both loops resided in the vicinity of the substrate binding pocket and phosphorylated T77 was located in the loop of Y34‐G105 (Figure 2g), which exhibited considerable RMSF variation, we speculated the RMSF variation in T77‐phosphorylated IDH1 may contribute to greater conformational variations. In parallel, a comparison of the B factor revealed that the hinge‐bending area (A74‐K93) was markedly altered in T77‐phosphorylated IDH1 (Figure S2g, Supporting Information, right panel) but not in non‐phosphorylated IDH1 (Figure S2g, Supporting Information, left panel). We further analyzed the variation in substrate binding pocket. The orientation of the carboxyl group in ICT changed dramatically in complex with T77‐phosphorylated IDH1compared to non‐phosphorylated IDH1; this created a more “open” state in the substrate binding area, facilitating the access of ICT to the active site (Figure 2h). In accord with the abovementioned results, the binding energy of non‐phosphorylated and T77‐phosphorylated IDH1 to ICT calculated by molecular mechanics‐generalized Born surface area (MM‐GBSA) method were −42.418 and −54.047 kJ mol^−1^ respectively (Figure 2i), indicating T77‐phosphorylated IDH1 binds to ICT with a higher affinity than its non‐phosphorylated counterpart does. Collectively, our data suggest that IDH1 T77 phosphorylation induces conformational changes that facilitate substrate binding and thus enhancing its activity.

### 2.3 IDH1 T77 Phosphorylation Diminishes Progestin Sensitivity in Endometrial Cancer

**Figure 3:**
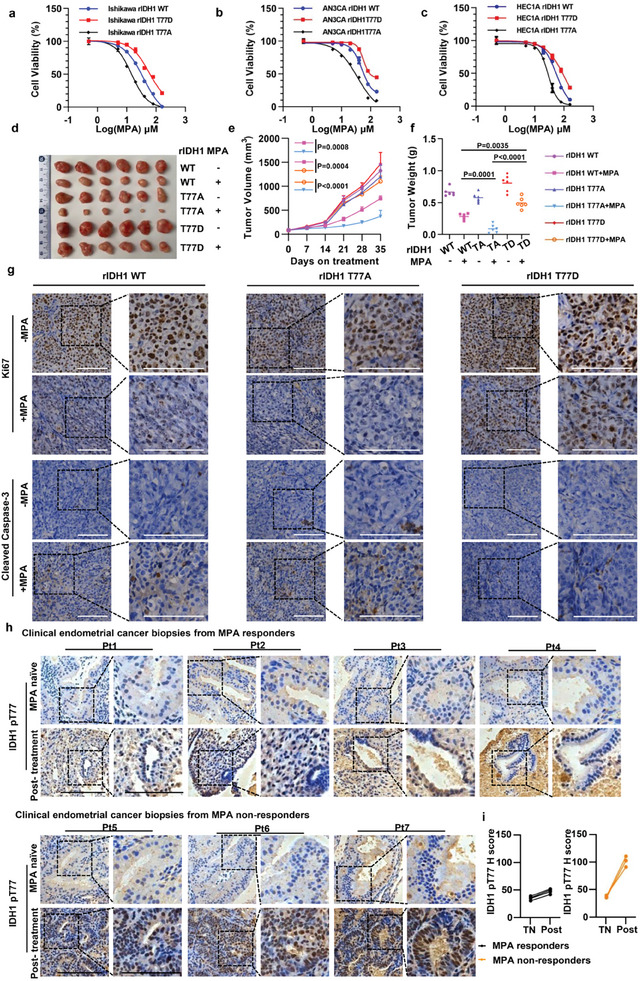
IDH1 T77 phosphorylation provides a growth advantage under MPA treatment and contributes to MPA resistance. a–c) Ishikawa (a), AN3CA (b) and HEC1‐A(c) cells harboring rIDH1WT or its TA/TD mutants were treated with increasing concentrations of MPA for 48 h. CCK8 analyses were performed. d) Tumors of Ishikawa rIDH1WT/TA/TD xenografts treated with vehicle or MPA were shown. e) Tumor volumes were shown. The values are presented as mean ±SD (*n* = 6 per group). The statistical test is based on 2‐way ANOVA combined with a Tukey‐corrected multiple‐comparison test. f) Tumor weight was shown. The values are presented as mean ±SD (*n* = 6). The statistical test is based on 1‐way ANOVA combined with a Tukey‐corrected multiple‐comparison test. g) IHC staining for Ki‐67 and Cleaved Caspase‐3 in the tumor sections from the indicated treatment groups was shown. Scale bar, 100 µm. h) Human endometrial cancer biopsies collected at baseline or after progression on MPA therapy were stained for pT77‐IDH1 and relative pT77‐IDH1 expression (H Score) from treatment naïve (TN) and post‐treatment (Post) was shown in i). Scale bars, 100 µm.

To further evaluate the role of T77‐phosphorylated IDH1 in progestin therapy, we expressed Flag‐tagged shRNA‐resistant (r) IDH1 variants in Ishikawa, AN3CA and HEC1‐A cells (Figure S3a, Supporting Information), and examined their sensitivity to MPA using CCK8 cytotoxicity assays. The IDH1 T77A mutation notably attenuated endometrial cancer cell viability upon MPA exposure and displayed half maximal inhibitory concentration (IC_50_) values much lower than those of WT IDH1 control cells. In addition, cells bearing the IDH1 T77D mutation were resistant to MPA (**Figure** **3**a–c; Figure S3b, Supporting Information). Next, we subcutaneously introduced Ishikawa cells expressing IDH1variants into nude mice to test their in vivo responsiveness to MPA. MPA treatment markedly impaired tumor volume (Figure 3d,e), weight (Figure 3f), and proliferation (Figure 3g) in the IDH1T77A tumor‐bearing group; however, in IDH1 T77D mice, no significant reduction in tumor growth was observed (Figure 3d–g). We next explored whether there was a correlation between pT77‐IDH1 levels and the response to MPA in patients with endometrial cancer. Sustained IDH1 T77 phosphorylation was observed in MPA‐resistant endometrial specimens, compared to that in the MPA‐sensitive group (Figure S3c–g and Table S10, Supporting Information). IHC staining also showed that the phosphorylation levels of IDH1 T77 were positively correlated with phosph‐p38 expression levels (Figure S3h,i, Supporting Information). We also investigated pT77‐IDH1 expression by immunohistochemical analysis of paired biopsies (treatment naïve and post relapse/complete response) from patients with EC who received MPA therapy (Table S11, Supporting Information). We observed that endometrial cancers that developed MPA resistance had significantly increased pT77‐IDH1 positivity (Figure 3h,i), and phosph‐p38 was markedly up‐regulated in MPA‐refractory clinical samples (Figure S3j,k, Supporting Information). Thus, our data highlight the importance of IDH1 T77 phosphorylation in MPA sensitivity.

### 2.4 Phosphorylation of IDH1 T77 Facilitates its Nuclear Translocation upon Progestin Exposure

**Figure 4:**
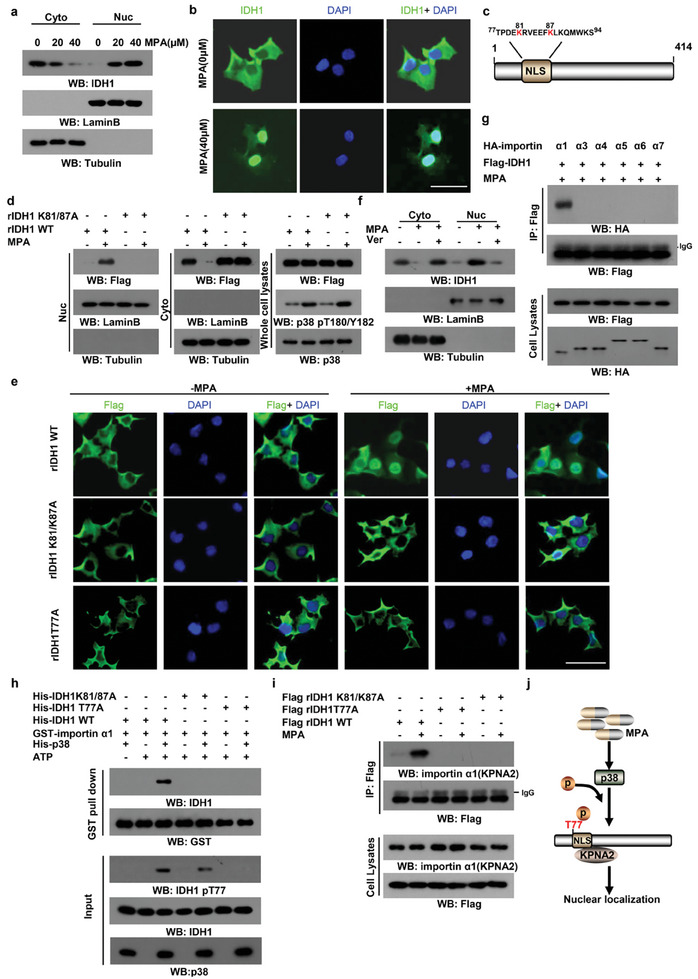
IDH1 nuclear redistribution is regulated by phosphorylation at T77. a) Cytosol and nuclear fractions derived from Ishikawa cells following MPA treatment were prepared. Immunoblot analyses were performed with indicated antibodies. b) Ishikawa cells were incubated with MPA. Immunofluorescent analyses were performed with an anti‐IDH1 antibody. Scale bar, 50 µm. c) Schematic of the predicted IDH1 NLS domain by NLStradamus and NLS Mapper tool. d) Ishikawa cells harboring WT IDH1 and its KA mutant were cultured with MPA. Cytosol and nuclear fractions were prepared for immunoblotting. e) Ishikawa cells expressing indicated Flag‐IDH1 proteins were treated with MPA or left untreated. Immunofluorescent analyses were performed with an anti‐Flag antibody. Scale bar, 50 µm. f) Ishikawa cells were treated with MPA and ivermectin or left untreated. Subcellular fraction assays were performed. g) Ishikawa cells harboring Flag‐IDH1 were co‐expressed with HA‐tagged importin‐α proteins under MPA treatment. Immunoprecipitation analyses were performed. h) Purified importinα‐1 was incubated with purified WT IDH1 or its TA and KA mutants in the presence of recombinant p38 and ATP. GST pull‐down analyses were performed. i) Ishikawa cells expressing WT IDH1 or its TA and KA mutants were incubated with or without MPA. Immunoprecipitation analyses were performed. j) A schematic diagram of IDH1 nuclear translocation in response to MPA.

We next sought to investigate how IDH1 T77 phosphorylation reduces progestin sensitivity in endometrial cancer cells. Posttranslational modifications of metabolic enzymes often lead to subcellular redistribution.^[^ ^29^ ^]^ Our aforementioned results indicated T77‐phosphorylated IDH1 was mainly located in the nucleus (Figure 3h). We thus postulated that progestin administration may result in IDH1 relocation to the nucleus. Cellular fractionation (**Figure** **4**a; Figure S4a, Supporting Information) and immunofluorescent (Figure 4b) assays showed that the proportion of IDH1 was increased in the nucleus following MPA treatment. Importin alpha targets classical nuclear localization signal (cNLS)‐containing proteins to link them to importin beta and mediates the nuclear translocation of target proteins. We therefore mutated Lys81/87 (K81/87) in the putative NLS sequences (Figure 4c; Figure S4b, Supporting Information) and analyzed the IDH1 location in response to MPA. Unlike WT IDH1, IDH1 K81/87A failed to translocate into the nucleus after MPA treatment (Figure 4d,e), indicating the importance of nuclear localization signals containing K81/87 for MPA‐dependent IDH1 nuclear import. In addition, MPA‐induced nuclear localization of the IDH1 T77A mutant was compromised (Figure 4e; Figure S4c, Supporting Information). Inhibition of importin‐α/β mediated nuclear translocation by Ivermectin^[^ ^30^ ^]^ largely blocked nuclear IDH1 (Figure 4f). We also found that MPA treatment resulted in the specific binding of IDH1 to importin‐α1 (IPOA1/KPNA2) (Figure 4g; Figure S4d, Supporting Information). Genetic silence of importin‐α1 by short hairpin RNA impaired MPA‐induced IDH1 nuclear transport (Figure S4e, Supporting Information). Furthermore, our in vitro results showed WT His‐IDH1 interacted with importin‐α1 in the presence of p38 and ATP; however, His‐IDH1 T77A or His‐IDH1 K81/87A (Figure 4h) or pharmacological inhibition of p38 (Figure S4f, Supporting Information) disrupted the interaction. Importantly, IDH1 T77A or IDH1 K81/87A mutant was unable to interact with importin‐α1 (Figure 4i), thereby raising the possibility that T77 phosphorylation facilitates the interaction between IDH1 and importin‐α1. In summary, our data demonstrate that phosphorylation of IDH1T77 by progestin exposes its NLS to importin‐α1 (KPNA2), which favors its nuclear translocation (Figure 4j).

### 2.5 IDH1 Couples with OCT6 to Regulate its Transcriptional Activity

**Figure 5:**
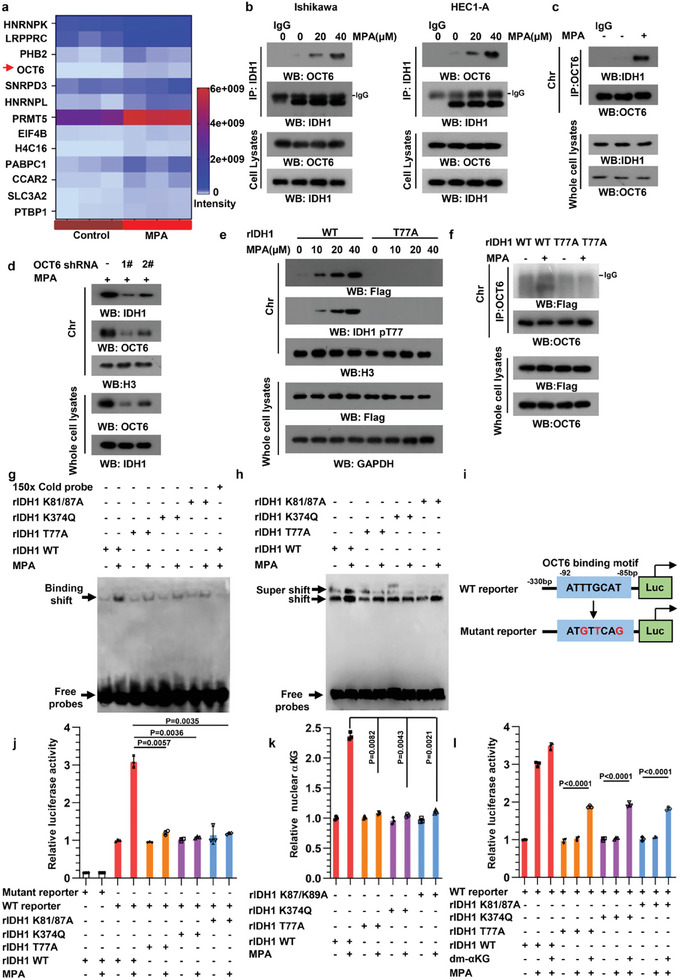
OCT6‐assciated IDH1 interacts with chromatin and mediates OCT6 transcription activity. a) Mass spectrometry analyses of IDH1‐associated nuclear proteins in Ishikawa cells treated with or without MPA for 24 h. The data were presented as a heatmap. OCT6 was identified to have strong interaction with IDH1 in response to MPA (red arrow). b) The interaction between IDH1 and OCT6 was confirmed by immunoprecipitation analyses with indicated antibodies. Affinity purified secondary antibodies that avoid IgG heavy‐chain interference were used when the membranes were incubated with a primary antibody against OCT6. c) Ishikawa cells were treated with MPA. Whole cell lysates and chromatin fractions were prepared. Immunoprecipitation analyses were performed with an anti‐OCT6 antibody. Affinity purified secondary antibodies that avoid IgG heavy‐chain contamination were used when the membranes were incubated with a primary antibody against OCT6. d) OCT6 was silenced in Ishikawa cells. Chromatin bound IDH1 was then detected by immunoblotting. e) Ishikawa WT/T77A IDH1 cells were treated with or without MPA. Chromatin fractions were prepared and chromatin‐bound total IDH1 and T77‐phosphorylated IDH1were detected by immunoblotting. f) Ishikawa WT/T77A IDH1 cells were treated with or without MPA. Chromatin fractions were prepared and immunoprecipitation analyses were performed. Affinity purified secondary antibodies that eliminate IgG heavy‐chain interference were used when the membranes were incubated with a primary antibody against OCT6. g) Ishikawa cells expressing IDH1 variants were treated with or without MPA. Nuclear extracts were prepared and an electrophoretic mobility‐shift assay (EMSA) was performed. h) Nuclear extracts derived from Ishikawa WT/TA/KA/KQ IDH1 cells were prepared. Electrophoretic mobility‐shift assays (EMSA) with an anti‐OCT6 antibody were performed. i) A schematic diagram to show the generated luciferase reporter plasmids. The plasmids contain the hH2BJ gene 5’‐flanking regions with an OCT6 binding motif (hH2BJ‐OCT6 WT) or a nonbinding control (hH2BJ‐OCT6 mutant). j) Ishikawa cells with IDH1 variants were transfected with pGL4.27‐hH2BJ‐OCT6 WT or mutant reporter, along with a pRL‐SV40‐Renilla plasmid internal control, and treated with or without MPA. The relative luciferase activity was detected (*n* = 3). The statistical test is based on 1‐way ANOVA combined with a Dunnett‐corrected multiple‐comparison test. k) Nuclei derived from Ishikawa WT/TA/KA/KQ IDH1 cells with indicated treatment were prepared and nuclear αKG was determined (*n* = 3). The statistical test is based on 1‐way ANOVA combined with a Dunnett‐corrected multiple‐comparison test. l) Ishikawa cells expressing IDH1 variants were cultured in the presence of MPA or cell membrane permeable dm‐αKG. The relative luciferase activity was detected (*n* = 3). The values are presented as mean ±SD by 2‐tailed Student's *t* test.

To further explore whether the nuclear redistribution of IDH1 could regulate gene transcription, we identified ≈400 binding partners of IDH1 in the presence of MPA using mass spectrometry (MS) and we focused our attention on nuclear proteins, especially the transcription factor. Among them, we found that only one transcriptional factor OCT6 (POU3F1), a mediator of the cellular response to doxorubicin,^[^ ^31^ ^]^ interacted with IDH1 in Ishikawa cells under MPA administration (**Figure** **5**a), which was further confirmed by immunoprecipitation analyses (Figure 5b). To determine whether phosphorylation of IDH1 T77 plays a role in IDH1/OCT6 complex formation, we constructed an IDH1 T77A mutant containing the NLS of SV40 large T‐antigen (Figure S5a, Supporting Information) and NLS‐IDH1 T77A resided in the nucleus regardless of MPA (Figure S5b, Supporting Information). Although WT IDH1 bound to OCT6 in response to MPA, IDH1 T77A or NLS‐IDH1 T77A disrupted the interaction (Figure S5c, Supporting Information). These data suggested that T77 phosphorylation is indispensable for the binding to OCT6. Notably, we found an association between IDH1 and OCT6 in chromatin following MPA treatment (Figure 5c), and OCT6 knockdown blocked chromatin‐bound IDH1 (Figure 5d). In conjunction with the finding that chromatin‐associated IDH1 T77A was reduced compared with its WT counterpart (Figure 5e), IDH1 T77A /OCT6 complex in the chromatin was diminished (Figure 5f), indicating T77 phosphorylation is crucial for the interaction of IDH1 and OCT6. We next tried to evaluate whether IDH1 affected OCT6 transcriptional activity following IDH1/OCT6 complex formation under MPA treatment. An electrophoretic mobility shift assay by incubation of the biotin‐labeled probe with nuclear extracts from IDH1 variant expressing cells showed MPA induced mobility complex formation in WT IDH1 cells; whereas the expression of IDH1 T77A and IDH1 K81/87A blocked it (Figure 5g). Importantly, the catalytically inactive IDH1 K374Q mutant which was able to translocate into the nucleus (Figure S5d, Supporting Information) also inhibited mobility complex formation, suggesting IDH1 catalytic activity is responsible for OCT6 transcriptional activity. These results were further supported by the super shift analysis (Figure 5h; Figure S5e, Supporting Information). We then generated wild‐type and point‐mutated luciferase reporter constructs containing the putative OCT6 binding motif of the hH2BJ (a known OCT6 target) proximal promoter (Figure 5i) and found an increase in reporter activity induced by MPA, which was blocked by IDH1 mutants (Figure 5j). IDH1 converts isocitrate to αKG. MPA treatment led to nuclear αKG accumulation in Ishikawa cells expressing WT IDH1; however, other IDH1 variants, including IDH1 T77A, IDH1 K81/87A and IDH1 K374Q did not (Figure 5k). Addition of cell membrane permeable dimethyl‐α ketoglutarate (dm‐αKG) rescued the reduced reporter activity by IDH1 mutants (Figure 5l). Taken together, our results indicate that OCT6 recruits nuclear IDH1 to the chromatin and that the catalytic activity of IDH1 is required for OCT6 transcriptional activity.

### 2.6 OCT6‐Associated IDH1 Targets Focal Adhesion under Progestin Therapy

**Figure 6:**
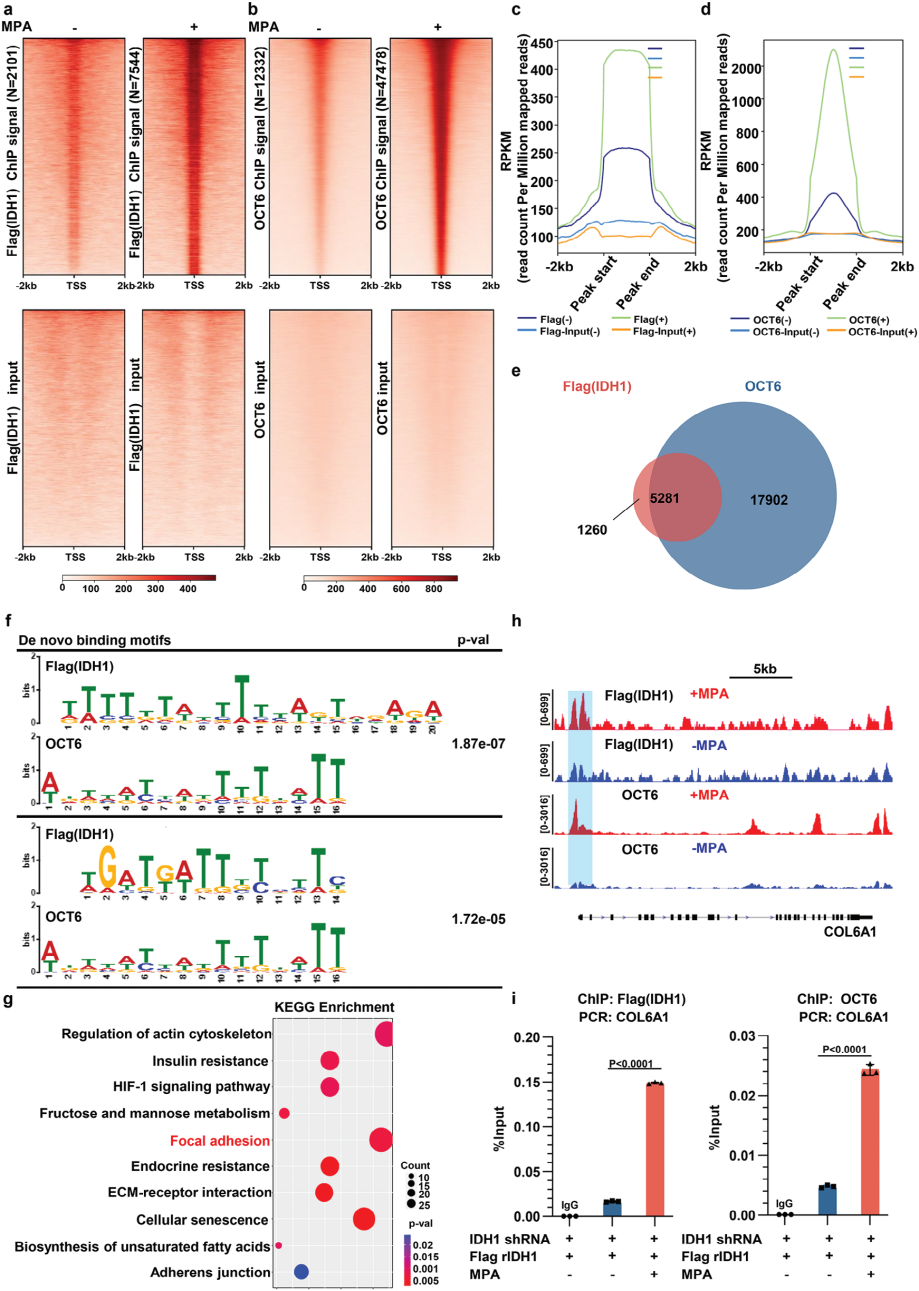
IDH1 and OCT6 coordinately regulate focal adhesion through increased chromatin occupancy in response to MPA. a,b) Ishikawa cells stably expressing Flag‐rIDH1 were treated with or without MPA. The chromatin was prepared for ChIP‐seq. Tornado plot representing Flag (IDH1) (a) and OCT6 (b) binding peaks in the presence or absence of MPA was shown. c) The aggregate plot of the RPKM of Flag (IDH1) ChIP‐seq data with the indicated treatments. d) The aggregate plot of the RPKM of OCT6 ChIP‐seq data with the indicated treatments. e)The Venn diagram representing the overlap of Flag (IDH1) and OCT6 target genes in Ishikawa cells treated with MPA. f) Selected homer de novo motifs of Flag (IDH1) and OCT6 were shown. g) KEGG analysis indicated Focal adhesion related genes were significantly enriched in the Flag (IDH1) and OCT6 target gene set where Ishikawa cells were treated with MPA. h) Genome browser views of Flag (IDH1) and OCT6 ChIP‐seq signals at COL6A1 gene loci with (red) or without (blue) MPA treatment. i) Ishikawa cells expressing Flag‐rIDH1 were incubated with or without MPA. ChIP analyses with anti‐Flag and anti‐OCT6 antibodies were performed (*n* = 3). The statistical test is based on an unpaired 2‐tailed Student's t test.

To determine the role of IDH1 and OCT6 in genome‐wide chromatin binding and identify critical downstream targets responsible for progestin responsiveness, we set out to perform chromatin immunoprecipitation sequencing (ChIP‐seq) in Ishikawa cells. However, this was limited by the lack of a commercial ChIP‐grade anti‐IDH1 antibody. Therefore, a ChIP‐seq analysis was conducted with the aforementioned Ishikawa cells expressing Flag rIDH1 WT using antibodies against Flag and OCT6. Following MPA administration, ChIP‐seq peaks for Flag (IDH1) and OCT6 were enriched in the promoter regions (Figure S6a, Supporting Information). Gene body enrichment analysis indicated that the peak signals of Flag (IDH1) and OCT6 were enriched at transcription starting sites (TSSs) and adjacent regions within 2 kb in response to MPA (**Figure** **6**a–d). The overall ChIP–seq profile of Flag (IDH1) binding was similar to that of OCT6 (Figure 6a–d; Figure S6a, Supporting Information). Eighty percent of the IDH1 target genes were also OCT6 targets (Figure 6e). We found the de novo motif of IDH1 and OCT6 highly overlapped (Figure 6f). We next performed Kyoto Encyclopedia of Genes and Genomes (KEGG) analysis to elucidate the gene loci that displayed increased chromatin binding of both IDH1 and OCT6 after MPA exposure. We found genes related to regulation of actin cytoskeleton, insulin resistance, HIF‐1 signaling pathway, fructose and mannose metabolism, focal adhesion, endocrine resistance, ECM‐receptor interaction, cellular senescence, biosynthesis of unsaturated fatty acid, and adhesion junctions were significantly enriched in both IDH1 (Flag) and OCT6 peaks upon MPA treatment (Figure 6g). among these pathways, HIF‐1 signaling pathway, adhesion junctions and focal adhesion have been reported to be activated in response to progestin in Ishikawa cells,^[^ ^32^ ^]^ highlighting the possibility that these pathways may regulate progestin sensitivity. Notably, focal adhesions are integrin‐dependent transmembrane complex which provide mechanical coupling between the surrounding extracellular matrix (ECM) and actin cytoskeleton. The multi‐protein focal adhesions facilitate cell communication with the surrounding ECM and play a crucial role in robust cell adhesion to the ECM. In tumor cells, aberrant cell ‐ECM interaction elicits altered signal transduction and often results in resistance to chemotherapy drugs or molecular agents, and is known as cell adhesion‐mediated drug resistance (CAM‐DR).^[^ ^33^ ^]^ IDH1 and OCT6 showed enhanced binding at the canonical focal adhesion target gene, COL6A1 (Figure 6h), which was further supported by the ChIP analysis (Figure 6i; Figure S6b, Supporting Information). In accordance with these findings, the expression of WT IDH1 upregulated the mRNA and protein levels of COL6A1 (Figure S6c,d, Supporting Information) under MPA treatment; however, the expression of the indicated IDH1 mutants largely blocked this effect. αKG, the metabolite of IDH1, acts as an epigenetic regulatory cofactor by mediating hydroxymethylation and histone methylation status at gene promoters.^[^ ^19a^ ^]^ We thus performed hMeDIP and ChIP assays and found increases in hydroxymethylation and decreases in tri‐methylation on H3K9 and H3K27 in the promoter region of COL6A1 in EC cells harboring WT IDH1 after MPA treatment (Figure S6e–g, Supporting Information). Taken together, our results suggest that progestin increases occupancy of IDH1and OCT6 at focal adhesion related gene loci.

### 2.7 Increased Focal Adhesion Reduces Progestin Responsiveness in Endometrial Cancer Cells

**Figure 7:**
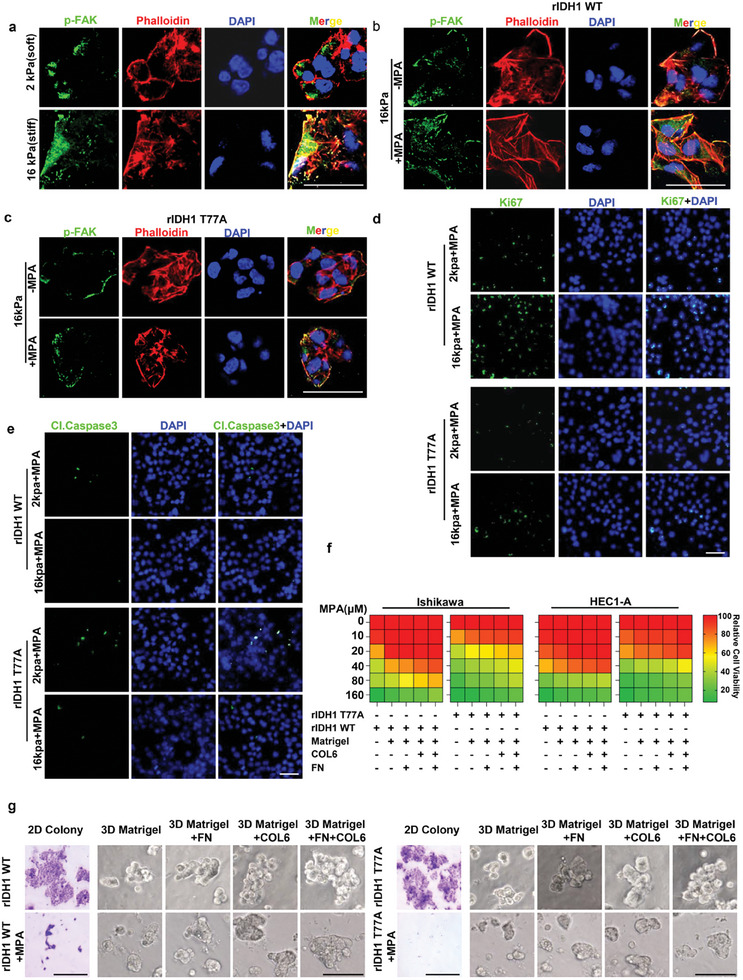
Increased focal adhesions confer resistance to MPA. a) Representative confocal micrographs of F‐actin (phalloidin, red) and phosphorylated focal adhesion kinase (p‐FAK, green) in cells on 2 and 16 kPa fibronectin‐functionalized polyacrylamide hydrogels. Scale bar, 50 µm. b,c) Ishikawa cells expressing WT IDH1 (b) or its T77A mutant (c) were seeded on 16 kPa fibronectin‐functionalized polyacrylamide hydrogels, followed by the administration of MPA. Representative confocal micrographs of F‐actin (phalloidin, red) and phosphorylated focal adhesion kinase (p‐FAK, green) were shown. Scale bar, 50 µm. d,e) Ishikawa cells with reconstituted expression of WT rIDH1 or rIDH1 T77A were cultured on 2 and 16 kPa fibronectin‐functionalized polyacrylamide hydrogels and treated with MPA. Representative confocal micrographs of Ki67 (d) and Cleaved‐Caspase 3 (e) were shown. Scale bar, 50 µm. f) Ishikawa and HEC1‐A cells expressing WT IDH1 or its T77A mutant were seeded in 96‐well plates coated with or without indicated ECM proteins, and treated with increasing concentrations of MPA for 48 h. CCK8 analyses were performed. g) Ishikawa cells expressing WT IDH1 or its T77A mutant were seeded in 2D condition (Scale bar, 500 µm) or 3D ECM matrices (Scale bar, 100 µm). The growth phenotype in response to MPA was shown.

We found IDH1 T77 phosphorylation diminished MPA sensitivity. Subsequent studies showed MPA‐induced IDH1 T77 phosphorylation promoted its nuclear translocation and interaction with OCT6; OCT6‐associated IDH1 bound to the promoter regions of focal adhesion related genes and facilitated their expression upon MPA treatment. These observations raised the possibility of a direct role of focal adhesion signaling in MPA responsiveness. ECM stiffness is considered to elevate focal adhesions,^[^ ^34^ ^]^ and we seeded Ishikawa cells on 2 and 16 kPa hydrogels and found enhanced stiffness increased focal adhesions (**Figure** **7**a). In addition, peripheral p‐FAK was also enhanced following MPA treatment in Ishikawa cells expressing WT IDH1 but not the IDH1 T77A mutant on stiff surface (Figure 7b,c), suggestive of increased focal adhesions upon MPA treatment. Ishikawa and AN3CA cells expressing IDH1variants were then cultured on soft and stiff hydrogels followed by MPA treatment. In endometrial cancer cells bearing WT IDH1, the proliferation was higher and apoptosis was lower at enhanced stiffness, which was rescued by reconstituted expression of IDH1 T77A (Figure 7d,e; Figure S7a,b, Supporting Information). These data suggested that increased focal adhesions could reduce the sensitivity to MPA‐induced apoptosis. ECM components together with their concentrations also contribute to matrix stiffness and focal adhesion assembly.^[^ ^35^ ^]^ Ishikawa cells expressing WT IDH1 and the IDH1 T77A mutant were cultured on 2D Matrigel (rich in laminin)‐containing combination matrices or not, and we found addition of ECM substrates was more effective in regulating focal adhesions (Figure S7c, Supporting Information). Accordingly, endometrial cancer cells expressing WT IDH1 exhibited treatment resistance on 2D ECM proteins (Figure 7f). In contrast, expression of IDH1 T77A mutant rescued the negative MPA response (Figure 7f). In line with these findings, 3D ECM matrices increased MPA resistance in WT IDH1 Ishikawa cells, compared with 2D cultures without ECM protein supplementation (Figure 7g). In summary, our data indicate that increased focal adhesions could reduce MPA effectiveness in endometrial cancer cells.

### 2.8 Pharmacological Inhibition of p38 or Focal Adhesion Sensitizes Endometrial Cancer to Progestin Therapy

**Figure 8:**
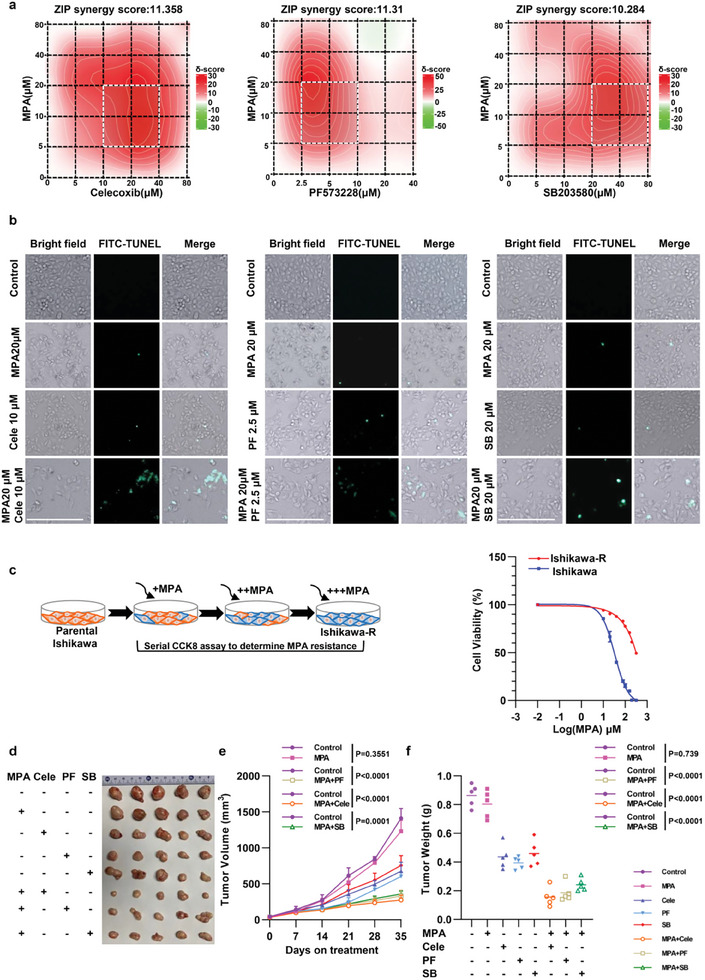
Pharmacological inhibition of p38 or focal adhesion improves MPA sensitivity. a) Ishikawa cells received monotherapy (including celecoxib, PF573228, SB203580 or MPA) and combination treatment. The drug interaction landscapes and the synergy score were analyzed by SynergyFinder 3.0. b) Ishikawa cells were cultured under the indicated treatment. FITC‐TUNEL analyses were performed. Scale bars, 250 µm. c) A schematic diagram (left) indicating the generation of MPA resistant Ishikawa cells (Ishikawa‐R). The parental and MPA‐resistant Ishikawa cells were treated with increasing concentrations of MPA for 48 h, and a CCK8 assay was performed (right). d) Tumors of Ishikawa‐R xenografts received indicated treatments were shown. e) Tumor volumes from different treatment groups were shown. The values are presented as mean ±SD (*n* = 5 per group) by a 2‐way ANOVA combined with a Tukey‐corrected multiple‐comparison. f) Tumor weight from different treatment groups was shown. The values are presented as mean ±SD (*n* = 5 per group) by a 1‐way ANOVA combined with a Dunnett‐corrected multiple‐comparison.

We next examined whether blocking of IDH1 T77 phosphorylation or focal adhesion could improve progestin responsiveness. Due to the lack of specific inhibitors targeting IDH1 T77 phosphorylation, the upstream kinase p38 inhibitor was utilized. Ishikawa and AN3CA cells were then treated with focal adhesion signaling inhibitors (Celecoxib^[^ ^36^ ^]^ and PF573228^[^ ^37^ ^]^) or a p38 inhibitor (SB203580)^[^ ^38^ ^]^ alone or in combination with MPA (**Figure** **8**a; Figure S8a, Supporting Information). We found MPA exhibited a highly synergistic effect with these agents, with the ZIP synergy scores of 11.358 (Celecoxib), 11.31 (PF573228), and 10.284 (SB203580), respectively (Figure 8a). Based on the calculated maximum synergistic area, the minimum effective concentrations of Celecoxib, PF573228, SB203580, and MPA were selected for further combination assays. Our results revealed that MPA, in combination with focal adhesion inhibitors or p38 inhibitor markedly induced apoptosis in endometrial cancer cells (Figure 8b), suggesting the suppression of focal adhesions or IDH1 phosphorylation improved MPA efficacy. Furthermore, we established an endometrial cancer cell line (Ishikawa) model with acquired MPA resistance (Figure 8c, left). The MPA‐resistant Ishikawa (Ishikawa‐R) cells were ≈tenfold more resistant as compared with its WT parental counterparts (Figure 8c, right) and the pT77‐IDH1 was elevated in Ishikawa‐R cells (Figure S8b, Supporting Information). The Ishikawa‐R cells were resistant to MPA, whereas combined therapy of MPA and focal adhesion inhibitors or p38 inhibitor exerted a robust cytotoxic response (Figure S8c, Supporting Information). The combined therapeutic effects were next evaluated in vivo. The control tumors expanded rapidly despite MPA, and single agents of Celecoxib, PF573228, or SB203580 exerted modest therapeutic efficacy. However, combinatorial treatment restored sensitivity to MPA (Figure 8d–f). Together, our data demonstrate that focal adhesion inhibition enhances the sensitivity of endometrial cancer cells to progestin.

## 3 Discussion

**Figure 9:**
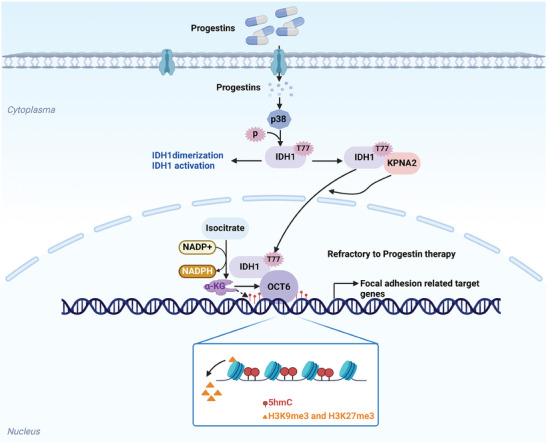
The schematic diagram illustrating the functions of IDH1 phosphorylation in progestin responsiveness. Upon progestin treatment, phosphorylated IDH1 by p38 translocates into the nucleus and is recruited to the focal adhesion related gene promoter via interaction with OCT6. Local αKG produced by IDH1 enhances OCT6 activity, decreases H3 methylation (H3K9me3 and H3K27me3) and increases hydroxymethylation level in target gene promoter, which transactivates focal adhesion signaling to confer progestin resistance. These findings unveil a negative feedback mechanism underlying a crucial role of IDH1 phosphorylation in regulating progestin sensitivity. The schematic diagram was created with BioRender (biorender.com).

High‐dose progestins are the current standard of care for patients with endometrial cancer who have the desire to preserve fertility.^[^ ^1a^ ^]^ However, progestin resistance commonly occurs.^[^ ^8^ ^]^ Albeit multiple molecular pathways related to progestin resistance have been reported, no effective predictive biomarkers have been approved.^[^ ^3b^ ^]^ In this study, we provide evidence that p38‐dependent IDH1 T77 phosphorylation confers progestin resistance by upregulating focal adhesion related gene expression (**Figure** **9**). Inhibition of IDH1 T77 phosphorylation or focal adhesion signaling may hold therapeutic potential to treat progestin‐resistant endometrial cancer.

The stress‐responsive p38MAPK (p38) kinases, are serine/threonine‐proline directed kinases activated by environmental stimuli, including oxidative stress, cytokines, infection, mechanical forces, cell damaging drugs, and hormones.^[^ ^39^ ^]^ Previous studies have indicated the role of p38 in progestin response by regulating progesterone receptor expression in peri‐implantation events.^[^ ^40^ ^]^ We found progestin treatment led to p38 activation in endometrial cancer cells; however, p38 activity is elevated by estrogen which acts as a proliferative stimulus in transformed epithelial cells of the female reproductive systems, and activated p38 induces ERα phosphorylation on T311 to promote its transcriptional activity by impairing ERα nuclear export and increasing the complex formation ability with its cofactors in endometrial cancer cells.^[^ ^41^ ^]^ This observation supports a role of p38 activity in stimulation of endometrial cancer cell growth. Therefore, in our present study, p38 activation and the subsequent IDH1Thr 77 phosphorylation may negatively regulate progestin sensitivity.

The posttranslational modifications of metabolic enzymes are responsible for their spatial and temporal regulation.^[^ ^29^ ^]^ IDH1 has been considered as a key metabolic enzyme that resides in the cytosol and peroxisome.^[^ ^17^ ^]^ Nonetheless, we identified a distinct IDH1 nuclear redistribution in response to progestin treatment. Specifically, progestin induced the p38‐dependent phosphorylation of IDH1 at T77 to expose its NLS for importin‐α1 (KPNA2) binding and the subsequent nuclear import. Nuclear IDH1 was recruited to the target genes promoter by the transcriptional factor OCT6, and its metabolite, αKG, in turn enhanced OCT6 activity and favored downstream gene transcription. This observation demonstrates that the non‐canonical nuclear function of IDH1 is controlled by T77 phosphorylation and this non‐canonical functional preference of IDH1 in response to progestin is integrated with its canonical role, which may endow endometrial cancer cells with the ability to adapt to multiple stressors in the tumor microenvironment. More recently, emerging evidence has revealed that metabolic enzymes participate in RNA splicing in the nucleus.^[^ ^42^ ^]^ Intriguingly, in our MS data, we found several RNA splicing‐associated proteins as binding partners of IDH1 under progestin treatment, suggestive of the potential role of nuclear IDH1 in RNA splicing. Future work is needed to explore how the RNA splicing and the canonical metabolic functions of IDH1 are coordinated to regulate progestin sensitivity.

Our finding that T77‐phosphorylated IDH1 level was upregulated in cell line models of resistance and MPA‐refractory specimens indicates a role for T77‐phosphorylated IDH1 in cases where progestin resistance has occurred and support the future testing of T77‐phosphorylated IDH1 targeting in endometrial cancer patients with progestin resistance. Moreover, in an attempt to identify effective biomarkers for the diagnosis of non–small cell lung cancer,^[^ ^43^ ^]^ He et al. demonstrated that the plasma levels of IDH1 were higher in non–small cell lung cancer patients and could be used as an auxiliary diagnostic marker. Therefore, IDH1 may function as a secretory protein and can be detected in circulation. In contrast to other tumors, little information is available regarding the indicative biomarkers in the peripheral blood of patients with endometrial cancer. Plasma T77‐phosphorylated IDH1 may be considered as a biomarker for identifying progestin therapy responsiveness.

Due to their upstream localization, focal adhesions play a critical role in regulating cell‐ECM adhesion. The aberrant adhesion of tumor cells to the ECM shields them from cytotoxic injury caused by chemotherapeutic drugs or molecular targeted agents, thus leading to the development of cell adhesion‐mediated drug resistance (CAM‐DR).^[^ ^33^ ^]^ As such, therapeutic strategies that impede focal adhesion maybe an ideal starting point for drug development. In this study, we found nuclear IDH1, in complex with OCT6, mutually facilitated the transcription of genes related to focal adhesion. We therefore identified a promising therapeutic combination for progestin‐resistant endometrial cancer. Repression of focal adhesion by pharmacological inhibitors re‐sensitized endometrial cancer cells to progestin therapy in vivo. However, inhibitors directly targeting wild‐type IDH1 or T77‐phosphorylated IDH1 are commercially unavailable. We thus suppressed p38, the upstream kinase of IDH1, to block IDH1T77 phosphorylation. As expected, p38 inhibition enhanced the responsiveness of endometrial cancer cells to progestin. These findings raise the possibility that the efficacy of progestin might be enhanced by the addition of focal adhesion inhibitors or T77‐phosphorylated IDH1 inhibitors, and highlight the potential applicability of these combined approaches. However, owing to the lack of direct inhibitor of IDH1T77 phosphorylation, the therapeutic effects of pT77‐IDH1 inhibition may be underestimated. The development of neutralizing antibodies or specific inhibitors against T77‐phosphorylated IDH1 will be needed in the future studies.

It is widely recognized that the efficacy of canonical cancer therapies could be improved by IDH1 inhibition.^[^ ^44^ ^]^ In pancreatic cancer cells, gemcitabine treatment induces HuR‐dependent IDH1 expression, and the HuR‐IDH1 regulatory axis in turn results in pancreatic cancer cell chemoresistance. Further mechanistic studies pinpoint ROS level and DNA damage as the critical factor for HuR‐IDH1 mediated chemoresistance and knockdown of IDH1 re‐sensitizes pancreatic cancer cell to gemcitabine.^[^ ^22b^ ^]^ Moreover, in a study aimed to illustrate the relation of IDH1 to glioblastoma biologic properties, researchers have found that IDH1 blockage enhances the responsiveness to targeted therapy by causing reduced αKG and NADPH, together with deficient lipid biosynthesis and an imbalance of redox homeostasis.^[^ ^22c^ ^]^ Many of the mechanisms of IDH1 in defense against anti‐ cancer drug‐induced cell death include a decrease in αKG, lipid synthesis and an increase in ROS and consequent DNA damage, all of which are likely related to its enzymatic activity. Thus, IDH1 activity may play a causal role in anti‐cancer drug sensitivity and resistance. We demonstrated progestin treatment led to the phosphorylation of IDH1 at T77, which contributed to the enhanced IDH1 activity. These observations support the notion that IDH1 T77 phosphorylation is closely associated with progestin sensitivity, which was further confirmed by our in vitro and in vivo study. Therefore, IDH1 T77 phosphorylation may render resistance to progestin therapy through its enzymatic activity‐associated effects, which awaits further investigation.

In conclusion, we report a previously unrecognized link between IDH1 T77 phosphorylation and progestin resistance in endometrial cancer. IDH1 T77 phosphorylation‐induced focal adhesion signaling creates therapeutic vulnerabilities in progestin resistance, and pharmacological blockage of IDH1 T77 phosphorylation or focal adhesion has the potential to be translatable into the clinical setting.

## 4 Experimental Section

### Cell Culture

Human endometrial cancer cell lines were obtained from ATCC and conserved in our lab. Ishikawa and AN3CA cells were cultured in Dulbecco's Modified Eagle Medium (DMEM, GIBCO) supplemented with 10% fetal bovine serum (FBS; GIBCO, Gaithersburg, MD, USA), 100 U mL^−1^ penicillin G and 100 µg mL^−1^ streptomycin (Life Technologies, Inc., Rockville, MD) in a humidified atmosphere of 5% CO_2_ at 37 °C. HEC1‐A cells were grown in RPIM‐1640 with 10% FBS. The MPA‐resistant Ishikawa cells were generated and grown in DMEM with 10% FBS. All cells were passaged with 0.25% trypsin/ EDTA in PBS when they reached a confluency of ≈80%. All cell lines were routinely tested for mycoplasma infection.

### Generation of Acquired MPA‐Resistant Endometrial Cancer Cell Line

Parental Ishikawa cells were treated with increasing concentrations of MPA over a period of approximately 6 months. The resistance status at each dose was ascertained by calculating the half maximal inhibitory concentration (IC_50_) of MPA using CCK8 assays. At the end of the treatment period, the cell line generated (Ishikawa‐R) was ≈tenfold more resistant compared with its parental counterparts.

### Animal Models

Six‐week‐old female athymic nude mice were housed in the Animal Facility at Shanghai General Hospital.

For the human endometrial cancer cell line derived xenograft model, Ishikawa cells (1 × 10^6^) harboring IDH1 variants were subcutaneously inoculated into the female nude mice. After establishing the tumors, the mice were randomly divided into different groups and received control or MPA treatment (i.p., 100 mg kg^−1^, and every other day). The tumor volume was measured once a week.

For the human MPA‐resistant cell line derived xenograft model, Ishikawa‐R cells (1 × 10^6^) were subcutaneously injected into the female nude mice. For all treatment studies, the mice were randomly assigned to different treatment groups when the tumors became palpable.

### Clinical Samples

Unpaired endometrial cancer biospecimens were obtained from patients (most of them have grade I endometrioid endometrial cancer) who had treated with progestin‐based medications, at the Shanghai General Hospital. Matched clinical endometrial cancer specimens (grade I endometrioid endometrial cancer) were collected at baseline or after progression after progestin‐based medications. All patients were informed and had the opportunity to provide consent for the use of their personal data.

### Plasmids and Mutagenesis

Human IDH1 cDNA was amplified and cloned into the pLVX‐IRES‐puro vector with Flag tag, and pET‐28 vector. Importin‐α1, ‐α3, ‐α4, ‐α5, ‐α6, and ‐α7 cDNA were amplified and cloned into pcDNA3.0‐HA vector. IDH1 mutations were generated using PCR and verified by sequencing.

### Mass Spectrometry

For identifying the IDH1‐associated proteins, the Flag beads‐bound proteins were extracted using SDT lysis buffer (4% SDS, 100 mm DTT, 100 mm Tris‐HCl pH 8.0), digested with trypsin (Promega, at 50:1 ratio) overnight at 37 °C and analyzed by a Q Exactive Plus mass spectrometer coupled with Easy 1200 nLC (Thermo Fisher Scientific). Proteins were identified by comparing the fragment spectra with those in the UniProt protein database (https://www.uniprot.org/proteomes/UP000005640) using MaxQuant 2.0.1.0.

### Coimmunoprecipitation Analysis

Endometrial cancer cells under indicated treatments were lysed using NP40 lysis buffer (50 mm Tris‐HCl, pH 7.4, 0.5% NP‐40, 150 mm NaCl, 1 mm EDTA, 10% glycerophosphate, and a cocktail of phosphatase and protease inhibitors). Cell debris were cleared by centrifugation. For coimmunoprecipitations of endogenous proteins, the soluble fraction of the cell lysates was quantified by the Bradford method and incubated with the indicated antibodies overnight followed by incubation with protein A/G agrose beads for 4h at 4 °C. For coimmunoprecipitations of Flag‐tagged proteins, cell lysates were incubated with Flag‐conjugated M2 beads overnight at 4 °C. After four washes, the proteins coupled to the beads were boiled in loading buffer and analyzed by immunoblotting.

### Western Blot Assay

Briefly, the samples were subjected to SDS‐PAGE and transferred onto a 0.45 µm PVDF membrane (Millipore). The membranes were blocked with 5% non‐fat milk or BSA in PBS and probed with indicated primary antibodies.

### RT–qPCR

Total RNA was extracted using the TRIzol (Invitrogen) reagent and isolated with the RNeasy kit (Qiagen). One microgram of total RNA was used for first strand cDNA synthesis with the HiScript III 1st Strand cDNA Synthesis Kit (Vazyme). Quantitative PCR was performed in a StepOne Real‐Time PCR System (Applied Biosystems), using the SYBR Green Realtime PCR Master Mix (TOYOBO).

### In Vitro Kinase Assay

In brief, bacterially purified recombinant WT and mutant His‐IDH1 were incubated with GST‐p38 in kinase assay buffer containing 25 mm Tris‐HCl (pH 7.5), 5 mm β‐glycerophosphate, 2 mm dithiothreitol (DTT), 10 mm MgCl_2_ and 0.1 mm Na_3_VO_4_ and 50 µm ATP‐γ‐S with or without the p38 inhibitor SB203580 at 30 °C for 45 min. The samples were then incubated with 50 mm PNBM/5% DMSO for 1 h at 25 °C, and subjected to SDS‐PAGE. Proteins phosphorylated by ATP‐γ‐S were determined by an anti‐thiophosphate ester antibody.

### Protein Crosslinking Assay

Endometrial cancer cells expressing the IDH1 variants were treated with or without MPA for 24 h. Cells were then lysed with lysis buffer (40 mm HEPES pH 7.5, 150 mm NaCl, 0.1% NP‐40 and a cocktail of protease inhibitors) followed by centrifugation. The supernatants were incubated with 0.025% glutaraldehyde for 30 min at 37 °C and the reaction was terminated by addition of 50 mm Glycine. The samples were subjected to immunoblotting.

### IDH1 Enzymatic Activity Assay

In brief, endometrial cancer cells were treated with or without MPA for 24 h. WT and T77A variant proteins were purified by immunoprecipitation with Flag‐M2 beads. The beads were then washed and eluted with the Flag peptide. A total of 2 µg purified proteins were added to 100 µL reaction buffer (25 mmol L^−1^ Tris‐HCl (pH7.5), 10 mmol L^−1^ MgCl_2_, 5 mmol L^−1^ DTT, 0.5 mmol L^−1^ NADP+ (Sigma‐Aldrich) and 1 mmol L^−1^ isocitric acid). The IDH1 activity was determined by measuring the absorbance at 340 nm every 20 s for 15 min.

### αKG Quantification

Intracellular αKG was detected by using the αKG concentration assay kit (Biovision) according to the manufacturer's protocol. To determine the nuclear αKG, nuclei were prepared by a Nuclei Pure Prep Nuclei Isolation Kit (Sigma), and the αKG concentration was detected.

### Measurement of NADP ^+^/NADPH

NADP+/NADPH was determined by an NADP^+^/NADPH Assay Kit with WST‐8 (Beyotime) according to the manufacturer's guidelines.

### Expression and Purification of Recombinant Proteins

Full‐length His‐IDH1 WT, His‐IDH1 T77A, and His‐IDH1 T77D and glutathione S‐transferase (GST)‐importin‐α1were expressed in BL21 (DE3) bacteria. The cultures were incubated at 37 °C to an OD600 of ≈0.6 followed by treatment with 0.5 mm isopropyl β‐d‐1‐ thiogalactopyranoside (IPTG) at 16 °C overnight. The cell pellets were lysed by sonication. Soluble His‐tagged proteins were bound to HisSep Ni‐NTA Agarose Resin, and eluted with 250 mm imidazole, followed by desalting using a PD‐10 column. For GST‐tagged proteins, the lysates were loaded onto a GSTrap HP column (GE Healthcare Life Sciences) and eluted with 10 mm reduced glutathione. Protein purity was examined by Coomassie Brilliant Blue staining.

### Surface Plasmon Resonance (SPR)

SPR experiments were performed on Biacore 8K (GE Healthcare) at 25 °C using diluted HBS‐EP buffer (BR100669, Cytiva) as previously described.^[^ ^45^ ^]^ The WT and mutated IDH1 proteins were immobilized onto the same CM7 sensor chips using different channels. The final response after immobilization was approximately 30000 RU. Increasing concentrations (6.25 µm, 12.5 µm, 25 µm, 50 µm, 100 µm) of isocitrate acid were flowed over the surface for single cycle kinetic experiments. The response units were recorded and processed using the BIAcore Insight Evaluation Software (3.0.12.15655. GE Healthcare). The resulting data were fit to a 1:1 binding model.

### Molecular Dynamics (MD) Simulation

The crystal structure of the human IDH1 complex (PDB: 1T0L) was retrieved from the Protein Data Bank (www.rcsb.org/). The T77‐phosphorylated IDH1 was constructed in the xleap module. All non‐standard residues except the NADP, ICT and phosphate group were removed before the simulation. The MD simulation was performed using the Gromacs 2018.4 program under constant temperature and pressure with periodic boundary conditions. The Amber99SB all‐atom force field and TIP3P water model were applied. In the MD simulation process, all bonds involving hydrogen atoms were constrained using the LINCS algorithm, with an integration time step of 2 fs. The electrostatic interactions were calculated using the Particle‐mesh Ewald (PME) method. The non‐bonded interaction cutoff value was set to 10 Å and updated every 10 steps. The V‐rescale temperature coupling method was used to control the simulation temperature at 300 K, and the Parrinello‐Rahman method was used to control the pressure at 1 bar. First, the steepest descent method was used to minimize the energy of the two systems to eliminate close contact between atoms; then, 1 ns of NVT and NPT equilibrium simulations were carried out separately at 300 K. Finally, 60 ns of MD simulations were performed separately for the two systems, and conformations were saved every 10 ps. The simulation results were visualized using the built‐in programs of Gromacs and VMD.

### Subcellular Fractionation Assay

Fractionation was performed using a Nuclear and Cytoplasmic Protein Extraction Kit (Beyotime) according to the manufacturer's guidelines. The cells were washed with ice‐cold PBS, lysed with Cytoplasmic Protein Extraction Buffer A for 15 min and then with Cytoplasmic Protein Extraction Buffer B on ice. The homogenates were centrifuged after vigorous vortexing, and the supernatants were used as the cytoplasmic fraction. Nuclear Protein Extraction buffer was then added to the pellet by vigorous vortexing for 30 min, followed by centrifugation. The supernatants were collected as nuclear fraction. The nuclear and cytoplasmic proteins were used in immunoblotting.

### Immunofluorescence Assay

Cells were washed with PBS fixed in 4% PFA for 15 min, and permeabilized with 0.1% Triton X‐100 for 10 min. The non‐specific binding was blocked with 1% BSA for 30 min at room temperature. The cells were then incubated with the primary antibodies including anti‐Flag, anti‐FAK pY397, and anti‐Paxillin overnight at 4 °C.The cells were stained with secondary antibodies. Phalloidin‐TRICT conjugates were used to indicate F‐actin. Nuclei were counterstained with DAPI.

### GST Pull‐Down Assay

A total of 100 ng of GST fusion proteins were incubated with His‐tagged purified protein along with glutathione agarose beads in binding buffer (50 mm Tris‐HCl pH 7.5, 1% Triton X‐100, 150 mm NaCl, 1 mm dithiothreitol, 0.5 mm EDTA, 100 mm PMSF, 100 mm leupeptin, 1 mm aprotinin, 100 mm sodium orthovanadate, 100 mm sodium pyrophosphate, and 1 mm sodium fluoride) at 4 °C. The glutathione beads were then washed with binding buffer and the bound proteins were eluted with 2× Laemmli buffer prior to SDS‐PAGE.

### CCK8 Cytotoxicity Assay

CCK8 assays were conducted according to the manufacturer's instructions (Yeasen). In brief, a total of 5000 cells per well were seeded in 96 well plates 12 h prior to treatment. The cells were treated with the indicated agents for 48 h. CCK8 solution was added to a final concentration of 10%. Relative cytotoxicity was determined by measuring absorbance at 450 nm.

### Immunohistochemistry Analysis

Tissue specimens from mouse or human were fixed in 4% paraformaldehyde (PFA) for 24 h and then embedded in paraffin wax. After de‐paraffinization and antigen retrieval, the 3 µm sections were incubated with the primary antibodies. Sections were developed with DAB and counterstained with hematoxylin. The following antibodies were used at the indicated dilutions: anti‐Ki67 (1:1000), anti‐Cleaved Caspase3 (1:500), anti‐IDH1 pT77 (1:50), and anti‐p‐p38 (1:500).

### Luciferase Reporter Assay

The promoter region (‐330bp upstream of the transcription start site) of hH2BJ containing the OCT6 binding motif ATTTGCAT (WT) or a nonbinding control ATGTTCAG (mutant) was amplified and cloned into pGL4.27 plasmids (Promega). Endometrial cancer cells with IDH1 variants were transfected with pGL4.27‐hH2BJ‐OCT6 WT or mutant reporter, along with a pRL‐SV40‐Renilla plasmid (Promega) as an internal control, and treated with or without MPA. Relative luciferase activity was detected by a Dual‐Luciferase Assay Kit (Promega).

### EMSA Assay

Biotin‐labeled oligomers were incubated with the nuclear extracts (10 µg per sample) in reaction buffer (10 mm Tris–HCl, pH 7.5, 1 mm MgCl_2_, 0.5 mm EDTA, 50 mm NaCl, 0.5 mm DTT, 4% glycerol, 0.05 mg mL^−1^ poly(dI‐dC) ∙poly(dI‐dC)). The DNA‐protein complex was separated from the free oligonucleotides on 7.5 % native polyacrylamide gels. For the supershift analysis, an anti‐OCT6 antibody was added to the binding reaction before incubation with labeled probe.

### Chromatin Immunoprecipitation (ChIP) and ChIP Seq Assay

The Chromatin Immunoprecipitation (ChIP) assays were conducted according to the manufacturer's instructions (Cell signaling technology). Cells were fixed with 1% formaldehyde (final concentration) for 10 min at room temperature, and the crosslinking reaction was terminated with 0.125 m glycine. The cells were then harvested with PBS and lysed in buffers A and B. The lysates were centrifuged and the supernatants were removed. The nuclear pellet was incubated in ChIP buffer and sonicated to obtain DNA fragments of ≈500 bp. Diluted chromatin was incubated with the antibodies including anti‐Flag, anti‐OCT6, anti‐H3K9me3, and anti‐H3K27me3 overnight at 4 °C. ChIP‐Grade Protein G agarose beads were added and incubated for 2 h at 4 °C with rotation. The agarose beads were pelleted and treated with sequential low‐ and high‐salt wash. Beads‐bound chromatin was eluted by ChIP elution buffer and de‐crosslinked at 65 °C for 2h. The DNA was purified by a PCR purification kit. Real time PCR analyses were performed with specific primers using immunoprecipitated DNA as templates.

For ChIP Seq analysis, endometrial cancer cells were treated with or without MPA. After 24 h, cells were washed with PBS and crosslinked with 1% formaldehyde. The nuclei were extracted and sheared by sonication in ChIP buffer. The sheared chromatin was incubated with anti‐Flag and anti‐OCT6 the antibodies and protein A/G beads. The chromatin was de‐crosslinked and the DNA was purified. Libraries were generated and throughput sequenced using HiSeq 2500 (Illumina). Peaks were called using MACS2 over the respective ChIP‐input.

### hMeDIP Assay

The hMeDIP assays were performed as previously described.^[^ ^23^ ^]^ Briefly, the total DNA in endometrial cancer cells with IDH1 variants was extracted and disrupted to 200–600 bp via ultrasonication, followed by incubation with 5hmC antibody. The DNA fragments were pulled‐down and used to amplify the target gene promoter by real time PCR.

### Chromatin Isolation

Chromatin preparation experiments were conducted as previously described with modification.^[^ ^46^ ^]^ Cells were incubated with buffer A (10 mm HEPES pH 7.9, 10 mm KCl, 1.5 mm MgCl_2_, 0.34 m sucrose and 10% glycerol) supplemented with protease inhibitors before the addition of Triton X‐100 (0.1%). The lysates were centrifuged, and the pellet (nuclei) was lysed with buffer B (3 mm EDTA, 0.2 mm EGTA, 1 mm DTT, and protease inhibitor cocktail) for 30 min with vortexing followed by centrifugation. The pellets were then solubilized in RIPA buffer with 500 mm NaCl and spun down at 14 000 rpm at 4 °C for 10 min. The supernatants (enriched for chromatin) were used for coimmunoprecipitation and immunoblotting assays.

### Polyacrylamide (PAA) Hydrogel Preparation

The coverslips were activated with 3‐aminopropyltrimethoxysilane in isopropanol for a final concentration of 2% before treatment with 1% glutaraldehyde solution and extensive washes. Polyacrylamide (PAA) hydrogels with different stiffness were prepared according to the previous formulations and dropped onto the hydrophobic microscope slide.^[^ ^47^ ^]^ The coverslips were then placed on top of the droplet to ensure that the gel solution could coat the entire coverslip. For cell seeding, the gels were treated with Sulfo‐SANPAH (1 mg mL^−1^) which was activated by ultraviolet irradiation. The gels were then incubated with 50 µg mL^−1^ fibronectin for 3 h at room temperature.

### TUNEL Assay

The TUNEL assay was performed with the One Step TUNEL Apoptosis Assay Kit (Beyotime) according to the manufacturer's guidelines. Briefly, cells were fixed with 4% PFA for 15 min and permeabilized with 0.5% Triton‐X 100. Cells were then incubated with the mixture of TdT enzyme, fluorescent labeling buffer as well as TUNEL detection buffer for 30 min at 37 °C. Fluorescence was detected under a microscope at an excitation wavelength of 450–500 nm and an emission wavelength of 515–565 nm.

### Study Approval

The use of human biospecimens and data for research purposes was approved by the Institutional Ethics Board of Shanghai General Hospital (2022KY053) and conformed to the Helsinki Declaration and to local legislation, and the patients' approval was obtained prior to the research. All procedures of animal studies were approved by the Institutional Animal Care and Use Committee of Shanghai General Hospital (2021AW011).

### Statistical Analysis

Results are expressed as mean ± SD. Unpaired two‐tailed Student's *t* test was used for two‐group comparisons. One‐way ANOVA combined with Dunnett's correction was used to evaluate data consisting of 3 and more groups. Two‐way ANOVA combined with Tukey's correction was used to determine groupwise comparison in three or more repeatedly measured groups. All experiments were performed at least three times except the animal experiments. *P* values less than 0.05 were considered statistically significant. Statistical analyses were performed using GraphPad Prism version 8.

## Conflict of Interest

The authors declare no conflicts of interest.

## Author Contributions

Z.Z., T.Z., and J.L. designed the research study. J.L., Z.Q., Y.L., Q.X., and P.C. performed the research. Y.L., B.H., and Y.L. contributed essential reagents or tools. J.L., T.Z., Y.L., Q.X., and P.C. analyzed the data. J.L., T.Z., and Z.Z. wrote the paper. W.Z. contributed to the research design. W.Z. participated in the review and revision of the manuscript.

## Supporting information

Supporting Information

## Data Availability

The data that support the findings of this study are available from the corresponding author upon reasonable request.
