# Supplementary material for: Phosphorylation of IDH1 Facilitates Progestin Resistance in Endometrial Cancer
Source: Adv Sci (Weinh). 2024 Apr 6;11(23):2310208. doi: 10.1002/advs.202310208 (PMC11187910; doi:10.1002/advs.202310208)
Supplement: Supplementary file 1 — Supporting Information [file ADVS-11-2310208-s001.docx]

Supporting Information

**Phosphorylation of IDH1 Facilitates Progestin Resistance in Endometrial Cancer**

**Short Running Title:** IDH1phosphorylation imparts progestin resistance to endometrial cancer

Jingjie Li^1^*, Zuoshu Qin^1^, Yunqi Li^2^, Baozhu Huang^1^, Qimeng Xiao^1^, Peiqin Chen^1^, Yifan Luo^1^, Wenxin Zheng^3,4,5^, Tao Zhang^6^* and Zhenbo Zhang^1, 7^*

^1^ Precision Research Center for Refractory Diseases, Shanghai General Hospital, Shanghai Jiao Tong University School of Medicine, Shanghai, 201620, China.

^2^ Shanghai Institute of Hematology, State Key Laboratory of Medical Genomics, National Research Center for Translational Medicine, Ruijin Hospital, Shanghai Jiao Tong University School of Medicine, Shanghai, 200025, China.

^3^ Department of Pathology, University of Texas Southwestern Medical Center, Dallas, TX 75390, USA

^4^ Department of Obstetrics and Gynecology, University of Texas Southwestern Medical Center, Dallas, TX 75390, USA

^5^ Simon Comprehensive Cancer Center, University of Texas Southwestern Medical Center, Dallas, TX 75390, USA

^6^ Department of Orthopedics, Shanghai General Hospital, Shanghai Jiao Tong University School of Medicine, Shanghai, 200080, China

^7^ Reproductive Medicine Center, Department of Obstetrics and Gynecology, Tongji hospital, School of Medicine, Tongji University, Shanghai, 200065, China

Figures S1-S8

Figure S1 IDH1 is phosphorylated at T77 by p38 under MPA treatment.

Figure S2 T77 phosphorylation-driven IDH1conformational changes lead to its enhanced enzyme activity.

Figure S3 IDH1 T77 phosphorylation is increased in MPA-resistant endometrial cancer specimens.

Figure S4 IDH1 phosphorylation at T77 promotes its nuclear redistribution.

Figure S5 IDH1 T77 phosphorylation is indispensable for the binding to OCT6.

Figure S6 Nuclear IDH1 and OCT6 coordinately regulate focal adhesion related target genes.

Figure S7 Focal adhesion alters endometrial cancer cell response to MPA.

Figure S8 Blocking focal adhesion or p38 enhances endometrial cancer cell responsiveness to MPA.

Table S1-S2 Supplementary Materials

Table S3-S9 Oligonucleotide sequences used in the study.

Table S10-S11 Basic clinical information of unpaired/paired endometrial cancer specimens.


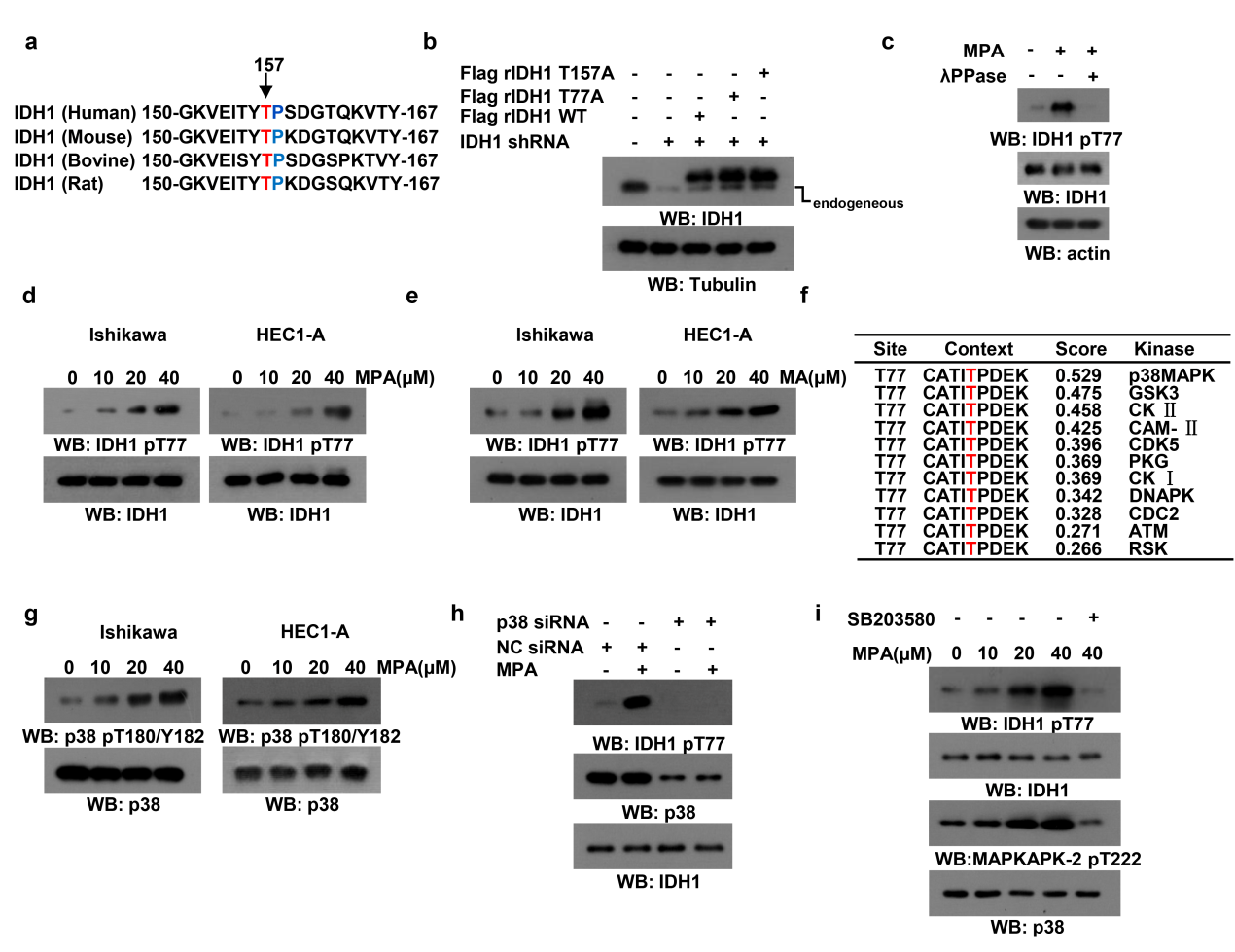


Figure S1

IDH1 is phosphorylated at T77 by p38 under MPA treatment. Related to Figure 1.

(a) T157 with its localization as part of T–P motif was indicated and highlighted in red. (b) Reconstituted expression of WT rIDH1, rIDH1 T77A, or rIDH1 T157A in Ishikawa cells with depleted endogenous IDH1. (c) Ishikawa cells were incubated with or without MPA. Cell extracts were prepared and treated with λPPase or left untreated. Immunoblotting analyses using indicated antibodies were performed. (d) Ishikawa and HEC1-A cells were treated with or without MPA for 24 h. Immunoblotting analyses were performed with indicated antibodies. (e) Ishikawa and HEC1-A cells were treated with or without MA for 24 h. Immunoblotting analyses were performed with indicated antibodies. (f) The putative upstream kinase of pT77-IDH1 was predicted by NetPhos 3.1. (g) Immunoblots of lysates from Ishikawa and HEC1-A cells treated with or without MPA. (h) Ishikawa cells were transfected with siRNAs targeting p38 and a non-specific control (NC). IDH1 and pT77-IDH1 levels were detected by immunoblotting. (i) Ishikawa cells were treated with increasing concentrations of MPA in the presence or absence of SB203580. Immunoblotting analyses were performed with indicated antibodies.


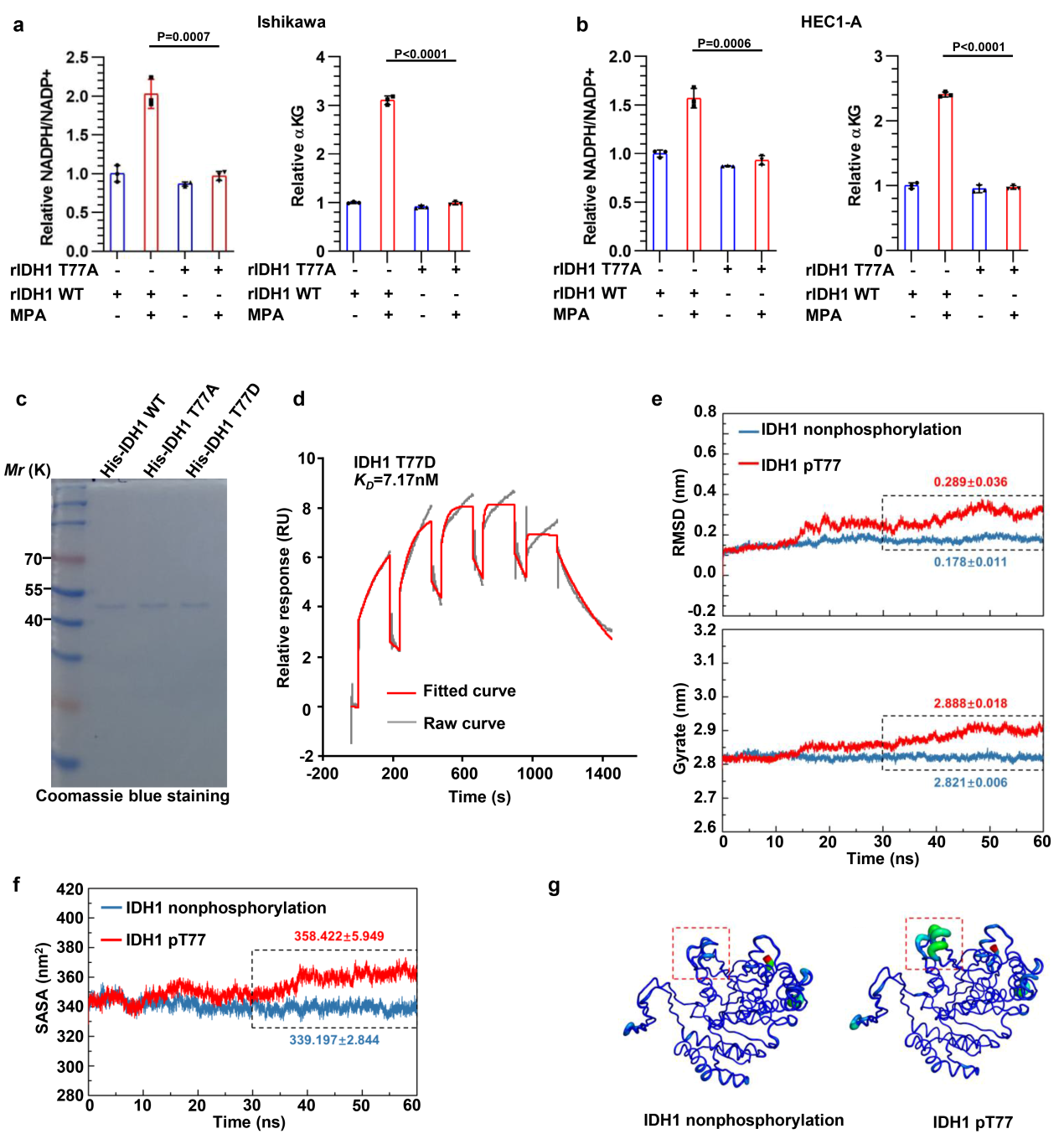


Figure S2

T77 phosphorylation-driven IDH1conformational changes leads to its enhanced enzyme activity. Related to Figure 2.

(a and b) Ishikawa (a) and HEC1-A (b) expressing indicated Flag-IDH1 proteins were treated with or without MPA. Cellular NADPH and αKG levels were determined. (c) Purified His-WT IDH1, T77A IDH1, and T77D IDH1 were subjected to SDS-PAGE followed by the Coomassie blue staining. (d) The SPR curves for the recombinant T77D IDH1 binding to ICT. (e) Evolution of the root mean square deviation (RMSD) and radius of gyration (Rg) for non-phosphorylated and T77 phosphorylated IDH1 in molecular dynamics simulations. Blue, non-phosphorylated IDH1; Red, T77-phosphorylated IDH1. (f) The solvent accessible surface area (SASA) for IDH1 with or without phosphorylation was shown. (g) B-factor structures of non-phosphorylated (left) and T77-phosphorylated IDH1 (right) based on molecular dynamics simulations. Red dashed box indicated highly fluctuated regions. In a and b, the values are presented as mean ±SD (n = 3) by a 2-tailed Student’s t test.


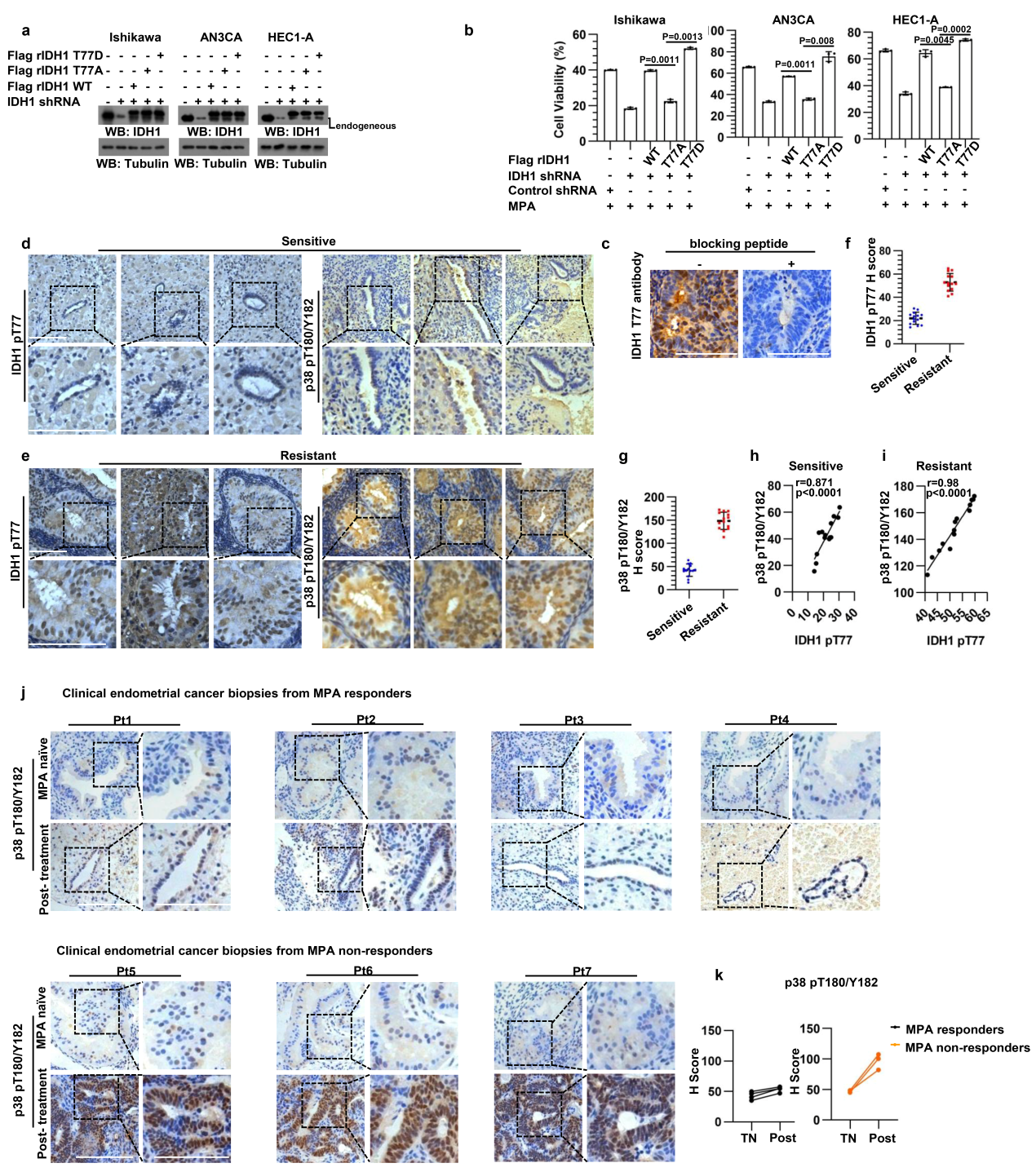


Figure S3

IDH1 T77 phosphorylation is increased in MPA-resistant endometrial cancer clinical specimens. Related to Figure 3.

(a) Endogenous IDH1 was depleted in Ishikawa, AN3CA, and HEC1-A cells. Cells were then infected with the lentivirus expressing Flag-tagged IDH1 WT, T77A, or T77D. Immunoblotting assays were performed. (b) Generation of indicated endometrial cancer cells with stable knockdown of endogenous human IDH1 and stable “rescue” expression of Flag-IDH1 WT, T77A, or T77D. Cell viability was determined by CCK8 assays. (c) Immunohistochemical staining of human endometrial cancer specimens from MPA non-responders was performed with the antibody against IDH1 pT77 in the presence or absence of the specific blocking peptide. Scale bars, 100 µm. (d and e) Representative IHC images of T77-phosphorylated IDH1 in unmatched MPA-sensitive (d) or resistant (e) specimens collected after MPA treatment. Scale bars, 100 µm. IHC staining scores were shown in (f and g). Pearson correlation test was used (h and i). (j) Representative IHC images of p38 pT180/Y182 in matched endometrial cancer specimens collected at baseline or after progression while on MPA therapy. Scale bars, 100 µm. (k) IHC staining scores of (j) were shown. In b, the values are presented as mean ±SD (n = 3) by a 1-way ANOVA combined with a Dunnett-corrected multiple-comparison test.


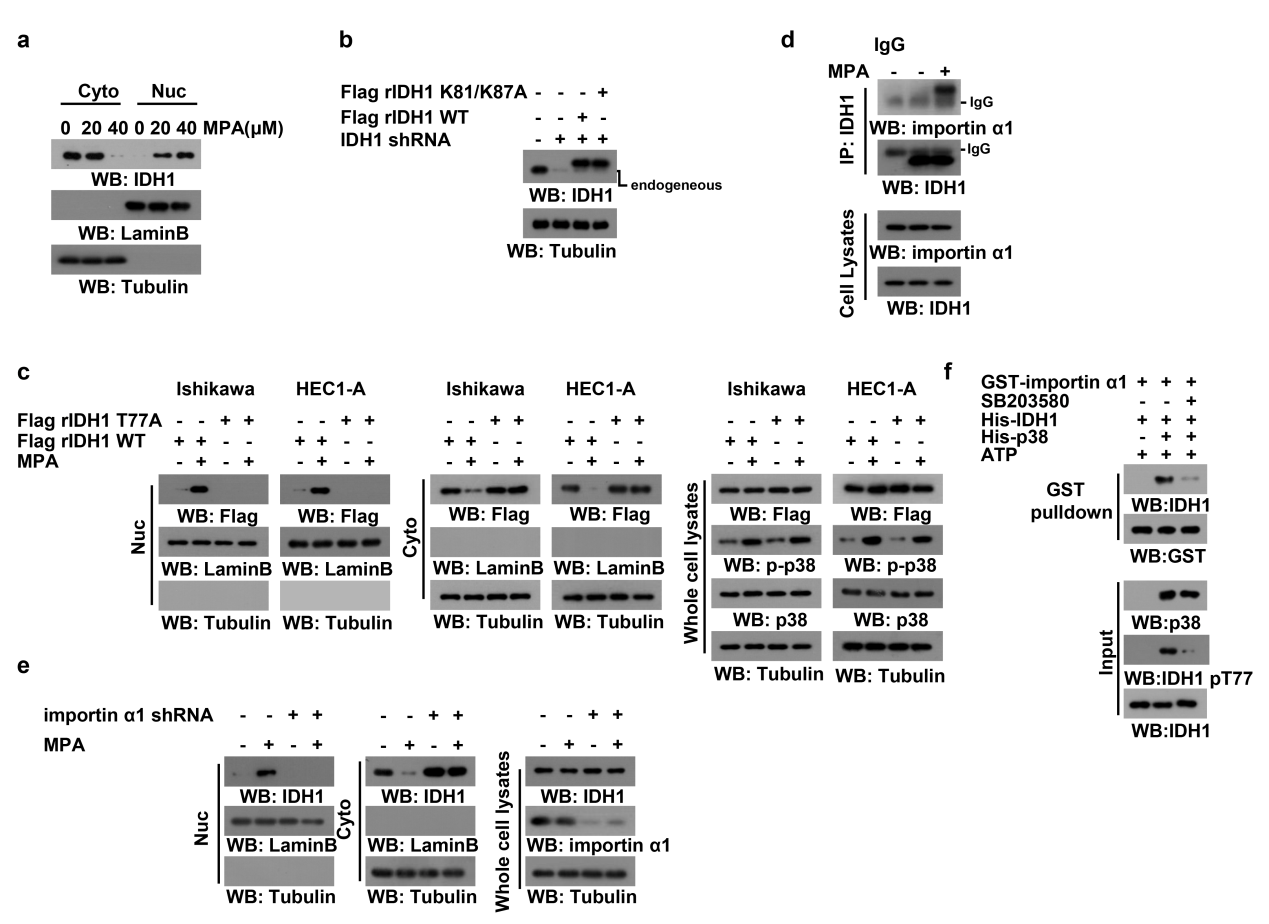


Figure S4

IDH1 phosphorylation at T77 promotes its nuclear redistribution. Related to Figure 4.

(a) Cytosolic and nuclear fractions in HEC1-A cells were prepared and immunoblotting analyses were performed. (b) Endogenous IDH1 was depleted in Ishikawa cells and cells were reconstituted with Flag-tagged IDH1 WT or K81/87A. (c) Ishikawa and HEC1-A cells expressing the indicated Flag-IDH1proteins were treated with or without MPA. Subcellular fraction assays were performed. (d) Ishikawa cells were treated with MPA or left untreated. Immunoprecipitation with an anti-IDH1 antibody was performed. (e) Cytosolic and nuclear fractions in Ishikawa or importin-α1 depleted Ishikawa cells with indicated treatment were prepared. Immunoblotting analyses were performed. (f) Purified importin-α1 was mixed with indicated His-IDH1 proteins in the presence or absence of p38 or SB203580. A GST pull-down assay was performed.


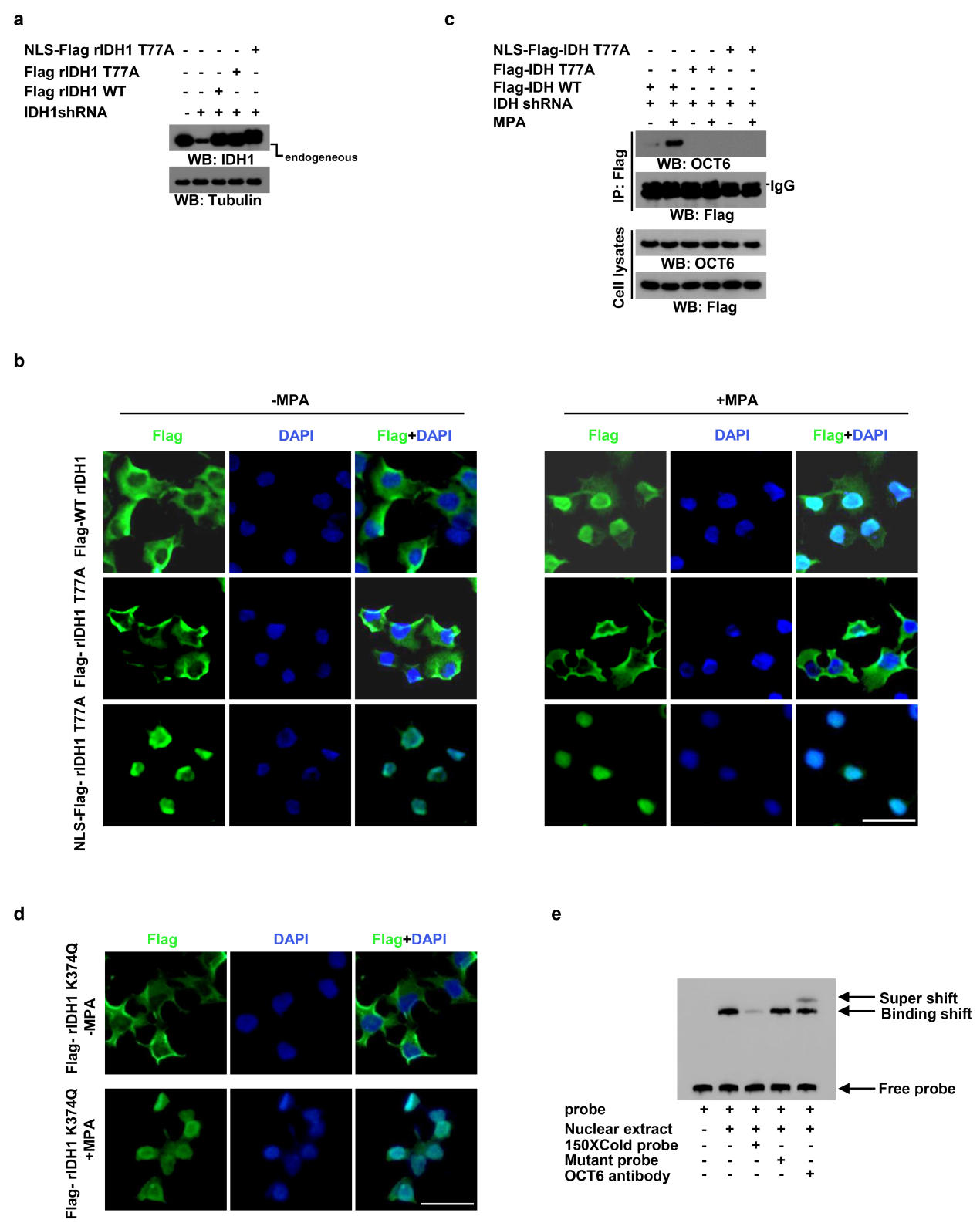


Figure S5

IDH1 T77 phosphorylation is indispensable for the binding to OCT6. Related to Figure5.

(a) IDH1-depleted Ishikawa cells with reconstituted expression of WT IDH1, IDH1T77A or NLS-IDH1 T77A were treated with or without MPA. An immunoblotting analysis was performed. (b) IDH1-depleted Ishikawa cells with reconstituted expression of WT IDH1, IDH1 T77A or NLS-IDH1 T77A were treated with or without MPA. An immunofluorescent analysis was performed. Scale bar, 50 µm. (c) Ishikawa cells expressing indicated Flag-IDH1 proteins were incubated with or without MPA. Immunoprecipitation analyses were performed using anti-Flag-M2 beads. (d) Ishikawa cells expressing the IDH1 acetylation-mimic K374Q mutant were treated with or without MPA and immunofluorescent analyses using an anti-Flag antibody were performed. Scale bar, 50 µm. (e) Nuclear extracts derived from Ishikawa cells expressing WT IDH1 were pre-incubated with the anti-OCT6 antibody followed by incubation with biotin-labeled WT, or mutant probe or a 150-fold excess of unlabeled WT probe. The protein–DNA complexes were separated by PAGE. Two retarded bands are indicated.


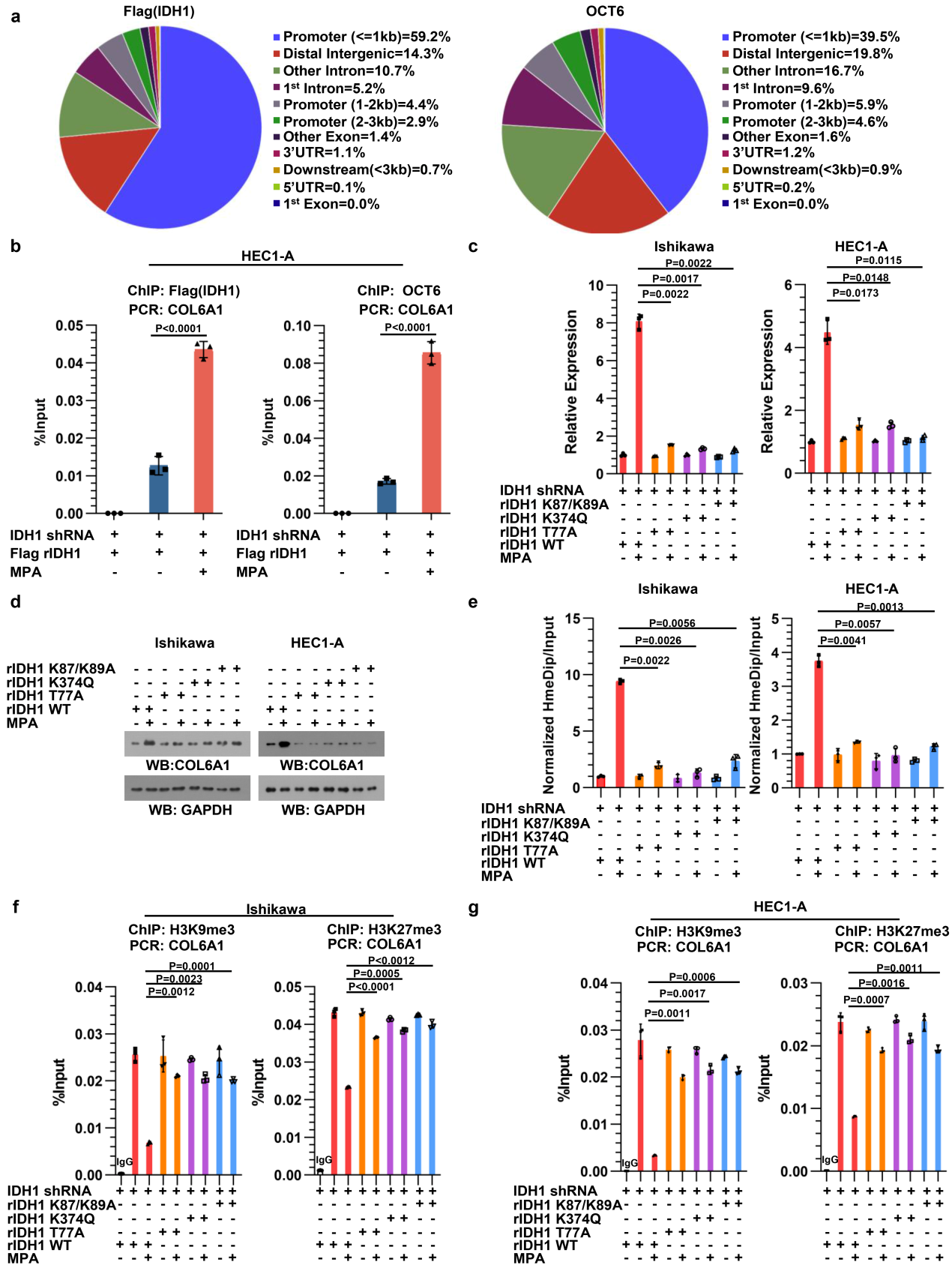


Figure S6

Nuclear IDH1 and OCT6 coordinately regulate focal adhesion-related target genes. Related to Figure6.

(a) The genomic distribution of Flag (IDH1) and OCT6 ChIP-seq peaks in Ishikawa cells post a 24 h MPA treatment. (b) HEC1-A cells expressing Flag-rIDH1 were incubated with or without MPA. ChIP analyses with anti-Flag and anti-OCT6 antibodies were performed. (c and d) Endometrial cancer cells harboring Flag-rIDH1 were treated with or without MPA. qPCR analysis of the mRNAs of the indicated gene (c) and immunoblotting analyses (d) with indicated antibodies were performed. (e) Total DNA in Ishikawa and HEC1-A cells harboring IDH1 variants received indicated treatment were subjected to the hMeDIP assay using an anti-5hmC antibody and primers flanking the COL6A1 promoter regions containing hydroxymethylated DNA fragments. (f and g) Ishikawa (f) and HEC1-A (g) cells harboring IDH1 variants were treated with or without MPA. ChIP assays were performed with indicated antibodies and amplified with primers targeting the OCT6 binding sites at the promoter regions of COL6A1. In b, the values are presented as mean ±SD (n = 3) by an unpaired 2-tailed Student’s t test. In c and e-g, the values are presented as mean ±SD (n = 3) by a 1-way ANOVA combined with a Dunnett-corrected multiple-comparison test.


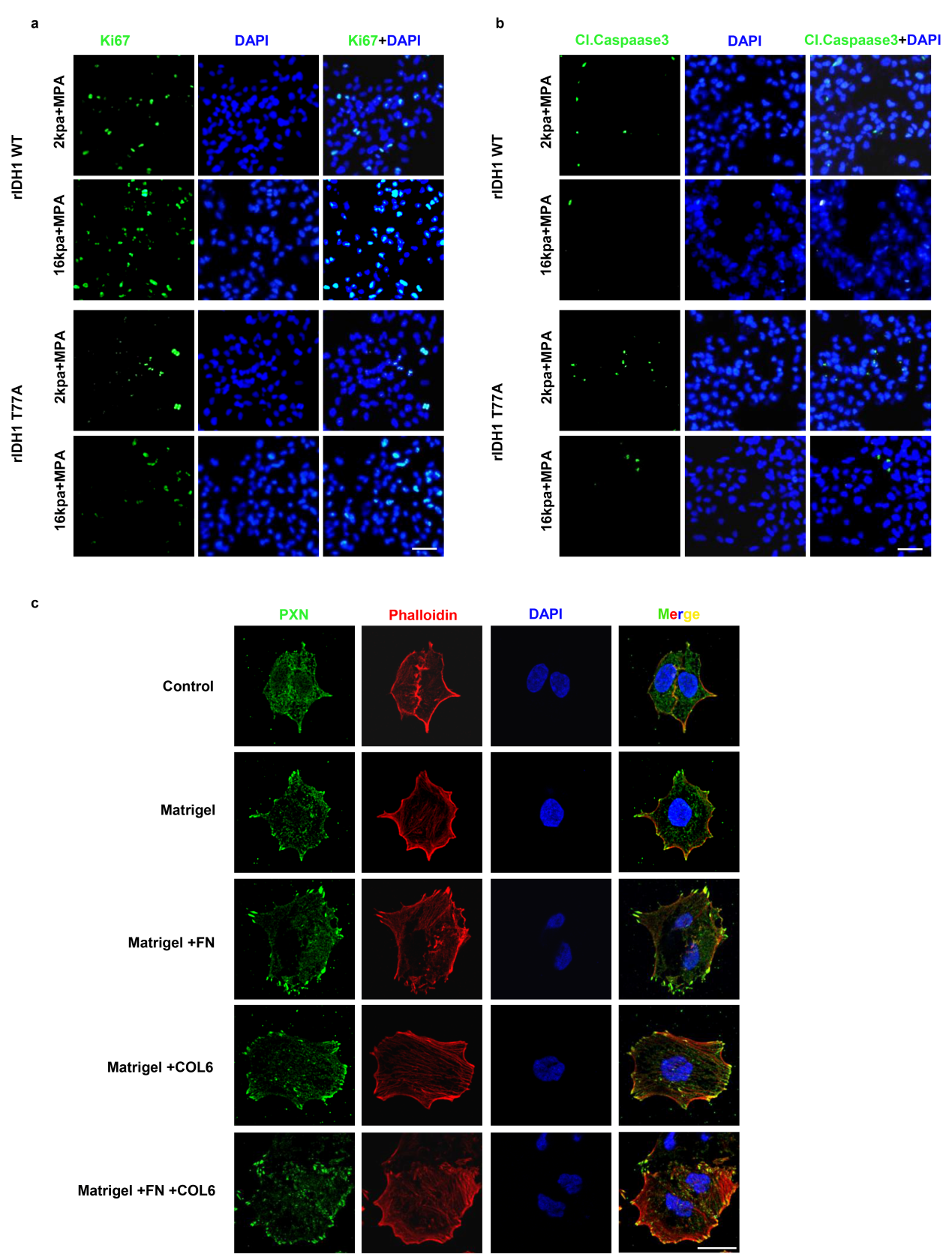


Figure S7

Focal adhesion alters endometrial cancer cell MPA response. Related to Figure7.

(a and b) Endogenous IDH1-depleted AN3CA cells with reconstituted expression of WT rIDH1 or rIDH1 T77A were cultured on 2 and 16 kPa fibronectin-functionalized polyacrylamide hydrogels. Representative confocal micrographs of Ki67 (a) and Cleaved Caspase 3 (b) were shown. Scale bar, 50 µm. (c) Ishikawa cells expressing WT IDH1 were cultured on indicated 2D matrices. Immunofluorescent analyses using an anti-Paxillin antibody were performed. Scale bar, 25 µm.


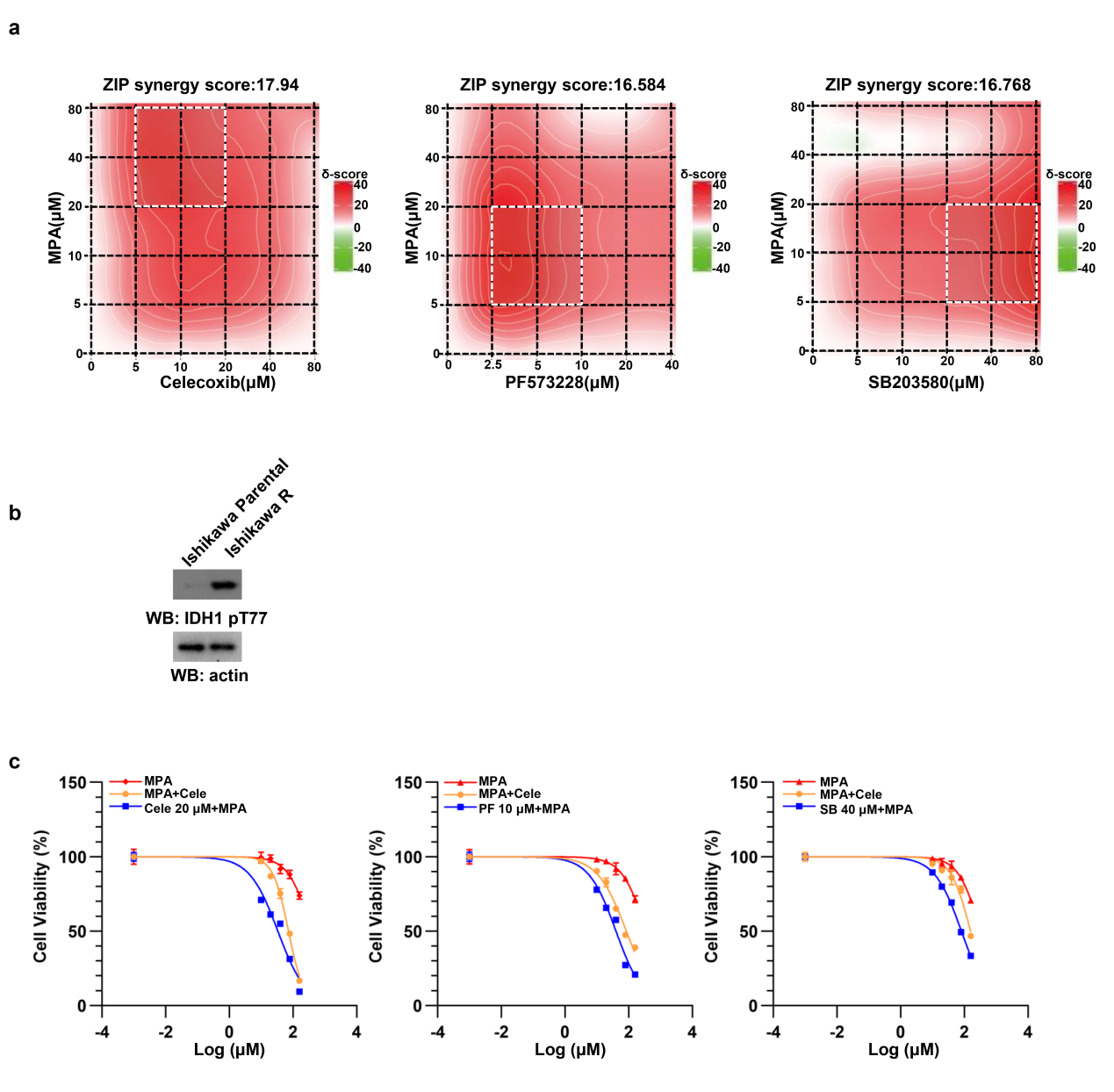


Figure S8

Blocking focal adhesion or p38 enhances endometrial cancer cell responsiveness to MPA. Related to Figure 8.

(a) AN3CA cells received monotherapy (including Celecoxib, PF573228, SB203580 or MPA) and combination treatment. The drug interaction landscapes and the synergy score were analyzed by SynergyFinder 3.0. (b) IDH1 pT77 protein level in Ishikawa and Ishikawa-R cells was detected by immunoblotting analyses. (c) Ishikawa and Ishikawa-R cells were treated in the presence or absence of MPA, focal adhesion inhibitors or a p38 inhibitor for 48 h. CCK8 assays were performed.

**Table S1 Antibodies used in this study.**

| **Antibodies** |
| --- |
| Anti-IDH1(Proteintech, 12332-1-AP) |
| Anti-Phosphothreonine-Proline / Phosphoserine-Proline (Abcam, ab9344) |
| Anti-Flag (Sigma, F1804) |
| Anti-Flag (Cell signaling technology, 14793) |
| Anti-β-Tubulin (Cell signaling technology, 2146) |
| Anti-Thiophosphate ester (Abcam, ab92570) |
| Anti-p38 (Cell signaling technology, 8690) |
| Anti-Phospho-p38 MAPK (Thr180/Tyr182) (Cell signaling technology, 4511) |
| Anti-Phospho-MAPKAPK-2 (Thr222) (Cell signaling technology, 3316) |
| Anti-His-Tag (Cell signaling technology, 12698) |
| Anti-GST (Abcam, ab111947) |
| Anti-HA-Tag (Cell signaling technology, 3724) |
| Anti-Ki67 (Cell signaling technology, 9449) |
| Anti- Cleaved-Caspase3 (Cell signaling technology, 9664) |
| Anti- Lamin B (Cell signaling technology, 13435) |
| Anti-KPNA2 (Cell signaling technology, 14372) |
| Anti-OCT6 (B-7) X (Santa cruz biotechnology, sc-376143X) |
| Anti-Histone H3 (Cell signaling technology, 4499) |
| Anti-FAK pY397 (BD Transduction Laboratories, 611722) |
| Anti-Paxillin (Proteintech, 10029-1-Ig) |
| Anti- Tri-Methyl-Histone H3 (Lys9) (Cell signaling technology, 13969) |
| Anti- Tri-Methyl-Histone H3 (Lys27) (Cell signaling technology, 9733) |
| Anti-5-Hydroxymethylcytosine (5-hmC) (Active Motif, 39769) |
| Anti-COL6A1 (Abcam, ab182744) |
| Anti-GAPDH (Cell signaling technology, 5174) |
| Anti-β-actin (Sigma-Aldrich, A1978) |
| Anti-Thiophosphate ester (Abcam, ab92570) |
| Alexa Fluor 488–conjugated anti–mouse IgG (Invitrogen, A21202) |
| Alexa Fluor 488–conjugated anti–rabbit IgG (Invitrogen, A21206) |
| Goat Anti-Mouse IgG HRP (eliminate heavy chain interference) (Abmart, M21004) |

**Table S2 Reagents used in this study.**

| **Reagents** |
| --- |
| Flag M2 agarose resin (Sigma-Aldrich, A2220) |
| Flag Peptide (Sigma-Aldrich, F4799) |
| Medroxyprogesterone acetate, MPA (MCE, HY-B0469) |
| dm-αKG (Sigma-Aldrich,349631) |
| Megestrol acetate, MA (MCE, HY-13676) |
| Alpha-Ketoglutarate Colorimetric Assay Kit (Biovision, K677-100) |
| Nuclei PURE Prep (Sigma-Aldrich, NUC201) |
| Nuclear and Cytoplasmic Protein Extraction Kit (Beyotime, P0028) |
| SimpleChIP® Enzymatic Chromatin IP Kit (Cell signaling technology, 9002) |
| ATP-γ-S (Abcam, ab138911) |
| DAPI (Thermo Fisher, R37606) |
| TRITC Phalloidin (Yeasen, 40734ES75) |
| NADP+/NADPH Assay Kit (Beyotime, S0179) |
| Cell Counting Kit-8 (Yeasen, 40203ES) |
| Dual-Luciferase Assay Kit (Promega, E1910) |
| One Step TUNEL Apoptosis Assay Kit (Beyotime, C1088) |
| 3-Aminopropyltrimethoxysilane (Sigma-Aldrich, 281778) |
| Sulfo-SANPAH (Sigma-Aldrich, 80332) |
| Growth factor-reduced Matrigel (Corning, 356231) |
| Human Collagen Type VI (Corning, 354261) |
| Human plasma-derived FN (Sigma-Aldrich，F2006) |
| DL-isocitric acid trisodium salt hydrate (Sigma-Aldrich, 1637-73-6) |

**Table S3 RNA interference (RNAi) sequences for knock-down experiments.**

| **Sequence name** | **Sequence (5’-3’)** |
| --- | --- |
| IDH1 shRNA | GCTGCTTGCATTAAAGGTTTA |
| KPNA2 shRNA | GCTGGTTTGATTCCGAAATTT |
| OCT6 shRNA-1# | CAAGAAGCGCACGTCCATCGA |
| OCT6 shRNA-2# | CACCACGCGCTGCACGAGGAT |
| p38 siRNA | AUGAAUGAUGGACUGAAAUGGUCUG |

**Table S4 Primers used for hH2BJ-OCT6 luciferase promoter cloning.**

| **Primer name** | **Sequence (5’-3’)** |
| --- | --- |
| WT reporter Forward | ACGTCCAGACATAGCGAGCG |
| WT reporter Reverse | CTTCGCTGGCTCTGGCATAG |

**Table S5 Primers used for site-directed mutation.**

| **Primer name** | **Sequence (5’-3’)** |
| --- | --- |
| IDH1 T77A Forward | CAAATGTGCCACTATCGCTCCTGATGAGAAGAG |
| IDH1 T77A Reverse | CTCTTCTCATCAGGAGCGATAGTGGCACATTTG |
| IDH1 T77D Forward | CAAATGTGCCACTATCGATCCTGATGAGAAGAGGG |
| IDH1 T77D Reverse | CCCTCTTCTCATCAGGATCGATAGTGGCACATTTG |
| Mutant reporter Forward | ACCTTATCTACCTAAGCGATTCTATATAAAAGCGCCT |
| Mutant reporter Reverse | CGCTTAGGTAGATAAGGTGAAGAGTTGAAGTCTTGTGTC |

**Table S6 Primers used for qPCR.**

| **Primer name** | **Sequence (5’-3’)** |
| --- | --- |
| COL6A1 Forward | ACAGTGACGAGGTGGAGATCA |
| COL6A1 Reverse | GATAGCGCAGTCGGTGTAGG |

**Table S7 Primers used for ChIP qPCR.**

| **Primer name** | **Sequence (5’-3’)** |
| --- | --- |
| COL6A1 promoter Forward | TACGTTCCAGCCTGGATGACA |
| COL6A1 promoter Reverse | TTCCACAACTCCACAGTGACTG |

**Table S8 Primers used for hMeDIP.**

| **Primer name** | **Sequence (5’-3’)** |
| --- | --- |
| COL6A1 promoter Forward | GATGAAAGTGCATGAAGGAA |
| COL6A1 promoter Reverse | GTGGCCACCGTGCTATAAGT |

**Table S9 Oligonucleotides for EMSA.**

| **Sequence name** | **Sequence (5’-3’)** |
| --- | --- |
| OCT6 probe | GCTCTTCACCTTATTTGCATAAGCGAT |

**Table S10 Basic clinical information of unpaired endometrial cancer patients**

| Case no. | Age (years) | Diagnosis | Grade | Post-progestin  findings |
| --- | --- | --- | --- | --- |
| Responders | | | | |
| 1 | 27 | ACH, localized endometrioid carcinoma | I | Progestin effects |
| 2 | 26 | ACH, localized endometrioid carcinoma | I | Progestin effects |
| 3 | 21 | endometrioid carcinoma | I | Progestin effects, SM |
| 4 | 28 | endometrioid carcinoma, localized ACH | I | Progestin effects |
| 5 | 34 | ACH, localized endometrioid carcinoma | I | Progestin effects |
| 6 | 33 | ACH, localized endometrioid carcinoma | Ⅱ | Focal ACH, SM, Progestin effects |
| 7 | 26 | endometrioid carcinoma, localized ACH | I | Progestin effects |
| 8 | 33 | ACH, localized endometrioid carcinoma | I | Progestin effects |
| 9 | 33 | endometrioid carcinoma, localized ACH | I | Progestin effects |
| 10 | 25 | ACH, localized endometrioid carcinoma | I | Progestin effects |
| 11 | 27 | ACH, localized endometrioid carcinoma | I | Progestin effects |
| 12 | 27 | endometrioid carcinoma | I | Progestin effects |
| 13 | 31 | ACH, localized endometrioid carcinoma | I | Progestin effects, SM |
| 14 | 27 | ACH, localized endometrioid carcinoma | I | Progestin effects |
| Non-Responders | | | | |
| 1 | 35 | APA, localized endometrioid carcinoma | I | APA, localized endometrioid carcinoma |
| 2 | 26 | ACH, localized endometrioid carcinoma | I | Focal ACH, localized endometrioid carcinoma |
| 3 | 33 | endometrioid carcinoma, localized ACH | I | Focal ACH, PE, localized endometrioid carcinoma |
| 4 | 27 | ACH, localized endometrioid carcinoma | I | Focal ACH, localized endometrioid carcinoma |
| 5 | 25 | endometrioid carcinoma | I | endometrioid carcinoma |
| 6 | 35 | endometrioid carcinoma | I | Focal ACH, localized endometrioid carcinoma |
| 7 | 29 | ACH, localized endometrioid carcinoma | I | Focal ACH, localized endometrioid carcinoma |
| 8 | 32 | endometrioid carcinoma | I | endometrioid carcinoma |
| 9 | 31 | ACH, localized endometrioid carcinoma | I | Focal ACH, localized endometrioid carcinoma |
| 10 | 24 | ACH, localized endometrioid carcinoma | I | Focal ACH, SM, localized endometrioid carcinoma |
| 11 | 34 | ACH, localized endometrioid carcinoma | I | Focal ACH, localized endometrioid carcinoma |
| 12 | 32 | endometrioid carcinoma | I | Focal ACH, SM, localized endometrioid carcinoma |
| 13 | 27 | ACH, localized endometrioid carcinoma | I | Focal ACH, localized endometrioid carcinoma |
| 14 | 35 | ACH, localized endometrioid carcinoma | I | Focal ACH, localized endometrioid carcinoma |

ACH, complex hyperplasia with atypia; APA, atypical polypoid adenomyoma; SM, squamous metaplasia; PE, proliferative endometrium.

**Table S11 Basic clinical information of paired endometrial cancer patients**

| Case no. | Age (years) | Diagnosis | Grade | Post-progestin  findings |
| --- | --- | --- | --- | --- |
| Responder | | | | |
| 1 | 32 | ACH, localized endometrioid carcinoma | I | Progestin effects |
| 2 | 29 | endometrioid carcinoma | I | Progestin effects |
| 3 | 31 | ACH, localized endometrioid carcinoma | I | Progestin effects |
| 4 | 24 | endometrioid carcinoma | I | Progestin effects |
| Non-responder | | | | |
| 1 | 39 | ACH, localized endometrioid carcinoma | I | Focal ACH, SM, localized endometrioid carcinoma |
| 2 | 21 | endometrioid carcinoma | I | endometrioid carcinoma |
| 3 | 28 | endometrioid carcinoma | I | endometrioid carcinoma |

ACH, complex hyperplasia with atypia; SM, squamous metaplasia.
